# Modulatory Effects of Sex Steroids Progesterone and Estradiol on Odorant Evoked Responses in Olfactory Receptor Neurons

**DOI:** 10.1371/journal.pone.0159640

**Published:** 2016-08-05

**Authors:** Ninthujah Kanageswaran, Maximilian Nagel, Paul Scholz, Julia Mohrhardt, Günter Gisselmann, Hanns Hatt

**Affiliations:** Ruhr-University Bochum, Department of Cell Physiology, Bochum, Germany; Monell Chemical Senses Center, UNITED STATES

## Abstract

The influence of the sex steroid hormones progesterone and estradiol on physiology and behavior during menstrual cycles and pregnancy is well known. Several studies indicate that olfactory performance changes with cyclically fluctuating steroid hormone levels in females. Knowledge of the exact mechanisms behind how female sex steroids modulate olfactory signaling is limited. A number of different known genomic and non-genomic actions that are mediated by progesterone and estradiol via interactions with different receptors may be responsible for this modulation. Next generation sequencing-based RNA-Seq transcriptome data from the murine olfactory epithelium (OE) and olfactory receptor neurons (ORNs) revealed the expression of several membrane progestin receptors and the estradiol receptor Gpr30. These receptors are known to mediate rapid non-genomic effects through interactions with G proteins. RT-PCR and immunohistochemical staining results provide evidence for progestin and estradiol receptors in the ORNs. These data support the hypothesis that steroid hormones are capable of modulating the odorant-evoked activity of ORNs. Here, we validated this hypothesis through the investigation of steroid hormone effects by submerged electro-olfactogram and whole cell patch-clamp recordings of ORNs. For the first time, we demonstrate that the sex steroid hormones progesterone and estradiol decrease odorant-evoked signals in the OE and ORNs of mice at low nanomolar concentrations. Thus, both of these sex steroids can rapidly modulate the odor responsiveness of ORNs through membrane progestin receptors and the estradiol receptor Gpr30.

## Introduction

The sense of smell is crucial for survival and important for socio-sexual communication in the majority of mammals. Furthermore, endocrine status is important for the physiological and behavioral adaptation of an organism. There is a growing body of evidence that blood circulating hormones and peptides have the ability to modulate the olfactory system [1–4]. There is a significant increase in sex hormones during pregnancy and the menstrual cycle; thus, these steroids likely play an important role in mediating behavioral and physiological changes.

Progesterone and estradiol mediate a wide range of physiological effects that go beyond female reproduction. Both steroids exert classical genomic actions and rapid non-genomic neuronal modifications through interactions with a diverse range of signaling molecules [5–7]. Several studies indicate that olfactory ability is differentially affected by hormonal changes based on the cyclical status of female rodents and humans [8–11]. According to a study by Pietras and Moultan (1974), estradiol increases olfactory sensitivity, and progesterone decreases the olfactory performance of female rodents in behavioral tests [9]. In humans, estradiol and progesterone appear to play a role in modulating olfactory sensitivity. During pregnancy, when the concentrations of estradiol and progesterone are dynamically altered, the evaluation of hedonic characteristics ascribed to odors changes, and women more frequently rate test odors as less pleasant smelling [12].

The presence of estradiol and mRNA for estradiol receptors in the olfactory bulb of rodents was reported [13,14]. Activation of these receptors can produce several actions, including the development of sexual dimorphism in olfactory pathways [15], processing of olfactory signals that mediate reproductive and social behavior [16–18] and olfactory memory formation [19]. While several studies have focused on estradiol and its impact on olfactory processing, little is known about the exact function of progesterone and the distribution of its putative receptors in the olfactory epithelium. Similar to estradiol, progesterone plays a role in many diverse functions, including the modulation of neuroplasticity, neuroprotection, neurogenesis, synaptic remodeling, cognitive functions and emotions [20–23].

The actions of progesterone are not only mediated via the classical nuclear progestin receptor (Pgr) but also via multiple novel progesterone receptors that have putative locations in the cell plasma membrane due to their rapid effects [24–26]. In contrast to the classical Pgr, these novel progesterone receptors act through rapid non-genomic actions rather than through slow genomic actions [27–30]. There are two groups of non-classical progesterone receptors: the membrane progestin receptors (mPRs) Paqr5-9 and members of the b5-like steroid-binding protein family, including progesterone receptor membrane component (Pgrmc) 1, Pgrmc2, neudesin and neuferricin, [25,26,31]. mPRs are composed of seven trans-membrane domains [32] and modulate cAMP levels via G-Proteins [33–35].

In a recent study, we showed that mPRs belong to the most highly expressed non-olfactory GPCRs in ORNs [36]. The strong expression of mPRs in ORNs suggests that progesterone can modulate odorant responsiveness through these receptors. Despite many reports that indicate changes in olfactory sensitivity fluctuate based on the circulating blood levels of ovarian steroid hormones, there is no evidence that progesterone or estradiol can directly modulate the odorant-evoked responses of ORNs in mammals. To our knowledge, we are the first to demonstrate that progesterone rapidly decreases the odorant-evoked responses of ORNs within one minute using submerged electro-olfactogram (EOG) and whole-cell patch clamp recordings of ORNs. Additionally, we showed that estradiol modulates odorant-evoked responses in a mechanism similar to that of progesterone. We detected the presence of proteins that are part of membrane progesterone and estradiol receptor proteins in the cilia of ORNs; therefore, progesterone and estradiol may rapidly modulate the OE through these receptors. Our results suggest that the levels of the female steroid hormones progesterone and estradiol have a direct modulatory action on the odorant-evoked responsiveness of ORNs.

## Materials and Methods

### Animals

C57BL/6J wildtype mice were obtained from Charles River (Sulzfeld, Germany). Transgenic OMP-GFP mice [37] and mOR-EG-GFP mice [38] were kindly provided by Dr. Peter Mombaerts and Dr. Kazushige Touhara. Mice were given normal laboratory chow and water ad libitum in standard cages. All animal experiments were carried out in accordance with the European Union Community Council guidelines and approved by the competent state office of the Federal Land of Northrhine Westphalia (file number 87–51.04.2010.A180).

### Submerged electro-olfactogram recordings

Submerged electro-olfactogram recordings were performed as previously described [39]. Adult mice were decapitated, the skin was quickly removed and their heads were cut along the sagittal plane. Subsequently, the dissected head was placed in low melting agarose to facilitate a transverse longitudinal position of the septum with the exposed OE. Using a custom made air-pressure application device, the OE was continuously perfused with oxygenated saline (0.6 ml/min) (oxygenated saline buffer: 120 mM NaCl, 25 mM NaHCO_3_, 5 mM KCl, 5 mM BES, 1 mM MgSO_4_, 1 mM CaCl_2_, and 10 mM glucose, pH 7.3, oxygenated with 95% O_2_, 5% CO_2_). The application cannula was placed on the calvaria with active suction at the maxilla. The reference electrode was embedded in the low melting agarose (4% in Ringer`s solution: 140 mM NaCl, 5 mM KCl, 2 mM CaCl_2_, 2 mM MgCl_2_, 10 mM HEPES, pH 7.3). The tip of the Ag/AgCl recording electrode pipette (2 MΩ) was sealed with agarose, filled with Ringer’s solution and placed directly on the surface of the epithelium. The application of incubated substances and odorants diluted in Ringer’s solution was controlled by a computer using 500 ms pulses for stimulation and an interstimulus interval of 4 min unless otherwise stated. The surface potentials were recorded and amplified with a DigiData 1200 Series interface (Axoninterface) and a DP 311 differential amplifier (WPI) and visualized with the program WinEDR V.3.1.2. (University of Strathclyde). One way AVOVA for repeated measurements (SigmaPlot, Systat GmbH, Germany) was used for statistical analysis of the amplitude and performed with the non-normalized data sets. Significant differences were calculated with the Tukey HSD Test (***: P<0.001, **: P<0.01, *: P<0.05).

### Whole Cell Patch-Clamp Recordings

Acute olfactory epithelium slices were electrophysiologically characterized using a recording chamber (Slice Mini Chamber, Luigs and Neumann). A confocal microscope (Leica DM 6000 CFS, Leica Microsystems) was used to visualize single neurons. A steel wired slice-hold-down (SHD26H/15, Hugo Sachs Elektronik, Harvard Apparatus GmbH) was used to fix the acute slices that were submerged with an oxygenated extracellular solution. Patch pipettes (6–9 MΩ) were pulled from borosilicate glass capillaries (GB150TF-8P, Science Products) using a PC-10 vertical two-step puller (Narishige Instruments) and fire-polished using a MF-830 Microforge (Narishige Instruments). Experiments were performed under optical control in the whole-cell configuration and recorded using an EPC-10 amplifier controlled by PatchMaster 2.20 (HEKA Elektronik, Lambrecht/Pfalz, Germany). The recording pipette solution contained 143 mM KCl, 10 mM HEPES, 1 mM EGTA, 2 mM KOH, 1 mM MgATP and 0.5 mM NaGTP (pH 7.1). The calculated liquid junction potential (JP-CalcW software) was automatically subtracted online. The pipette and cell membrane capacitance were automatically compensated for during the measurement. The experiments were performed by continuously applying an oxygenated extracellular solution at a holding potential of—56 mV. Odorants were applied using a pressure-driven microcapillary application system. Slices were used for only one measurement to exclude odorant contamination of untreated cells. Data were analyzed offline using IGOR Pro 6.05 (Wave Metrics, Portland, OR) with Neuromatic 2.0 (Jason Rothman) and Excel 2010 (Microsoft) software. A Student´s t-test was used for statistical analysis of the amplitude, and significant differences were calculated (**: P<0.01, *: P<0.05).

### RT-PCR

Mice were decapitated and the OE was collected from the septum and turbinates. Total RNA from single mouse samples was isolated using an RNeasy Mini Kit (Qiagen, Hilden, Germany) including a DNaseI digestion. cDNA was prepared using an iScript cDNA Synthesis Kit (Bio-Rad, München, Germany). PCR reactions (20 μl total volume, 1 min, 95°C; 1 min, 58°C; 1 min, 72°C; 40 cycles) were performed on a Mastercycler RealPlex (Eppendorf, Hamburg, Germany) using the GoTaq qPCR Master Mix (Promega, Madison, WI, USA). Primers for genes of interest were designed with Primer-BLAST (sequence 5’ to 3’; forward/reverse):

Paqr5 (GCCCCAGGTGTTCCATGAG; AGGCTGAAGAGATTGACGGC)

Paqr6 (CCCACCTGGTACTTCCTGTG; GAAAGAGTTGAGCGCAGCAG)

Paqr7 (GTGGCCGCTACCCCTTTATC; CTCAGGAAAACTTCTGATGGGC)

Paqr8 (CCGCGCTGGTGTTCGAGTT; CCCACTCATTGACAGGGTGC)

Paqr9 (CGTGGAGTGCTTCATCCTGT; TGCCGAAGCCATAGTAGCTG)

Pgrmc1 (GTTCTACGGGCCTGAGGGG; TTCTTCCGAGCTGTCTCGTC)

Pgrmc2 (CTTCCCCAACAGCTGCCTAA; CTGGCTCACGGCTCTGTATT)

Gpr30 (CCTCTGCTACTCCCTCATCG; ACTATGTGGCCTGTCAAGGG)

Esra1 (ACCATTGACAAGAACCGGAG; CAGAATAGATCATGGGCGGT)

Esra2 (GAAGCTGGCTGACAAGGAAC; GTGTCAGCTTCCGGCTACTC)

### Immunohistochemistry

Immunostaining of the OE was performed on 12 μm coronal cryosections of paraformaldehyde-fixed tissue from adult C57BL6 mice on superfrost slides (Thermo Scientific, Menzel Gläser). After blocking with 1% fish gelatin in phosphate-buffered saline containing 0.1% Triton X-100, sections were incubated with primary antibody (anti-Paqr8 (Abcam): 1:100, anti-Pacr9 (Abcam): 1:50, anti-GPR30 (Abcam): 1:100, anti-Pgrmc1 (Sigma-Aldrich): 1:100) and fluorescently-labeled secondary antibody (Invitrogen, anti rabbit Alexa 456 nm, 1:100) dilutions in blocking solution. Stained sections were mounted in ProLong Antifade Gold medium (Molecular Probes). All fluorescence images were collected on a confocal laser scanning microscope (LSM510 Meta, Zeiss, Oberkochen, Germany). For the anti-Pgrmc1 antibody, the staining was performed on tissue sections following a heat-induced epitope antigen retrieval using citrate buffer (10 mM citric acid, 0.05% Tween 20, pH 6.0). Control experiments completed without addition of the primary antibody revealed a low level of background staining.

### cAMP-Glo assay

The OE of 10 mice (5 male and 5 female) was prepared, pooled, and the epithelia were minced. Next, the cells were dissociated by adding papain as previously described [36]. The steroid hormones were applied with a preincubation time of 8 minutes. Subsequently, cells were exposed to Henkel 100 diluted in Ringer`s solution for 15 min. The cAMP level was measured using a cAMP-Glo^™^ Assay (Promega) according to the manufacturer’s instructions. All data were normalized to the negative control that consisted of cells treated with 0.1% DMSO in Ringer`s solution. One way AVOVA was used for statistical analysis of the data, and significant differences were calculated (***: P<0.001, **: P<0.01, *: P<0.05).

### Chemical substances

The odorants used in this study were provided by Sigma-Aldrich or Henkel AG (Düsseldorf, Germany). Dilutions of the odorant stock solutions in DMSO were freshly prepared before use and had a maximum DMSO concentration of 0.1%. RU-486, estradiol and progesterone were purchased from Sigma-Aldrich (St. Louis, MO, USA), and G15 was purchased from Tocris Bioscience (Briston, UK). The Henkel 100 mix (Henkel AG, Düsseldorf, Germany) is a complex odor mixture composed of 100 odorants. In the 1:5,000 dilution which is typically applied, the approximate total concentration of odors is 1 mM or 10 μM for every single odorous substance, repectively.

## Results

### Expression pattern of steroid hormone receptors in the OE based on RNA-Seq data

**Fig 1:**
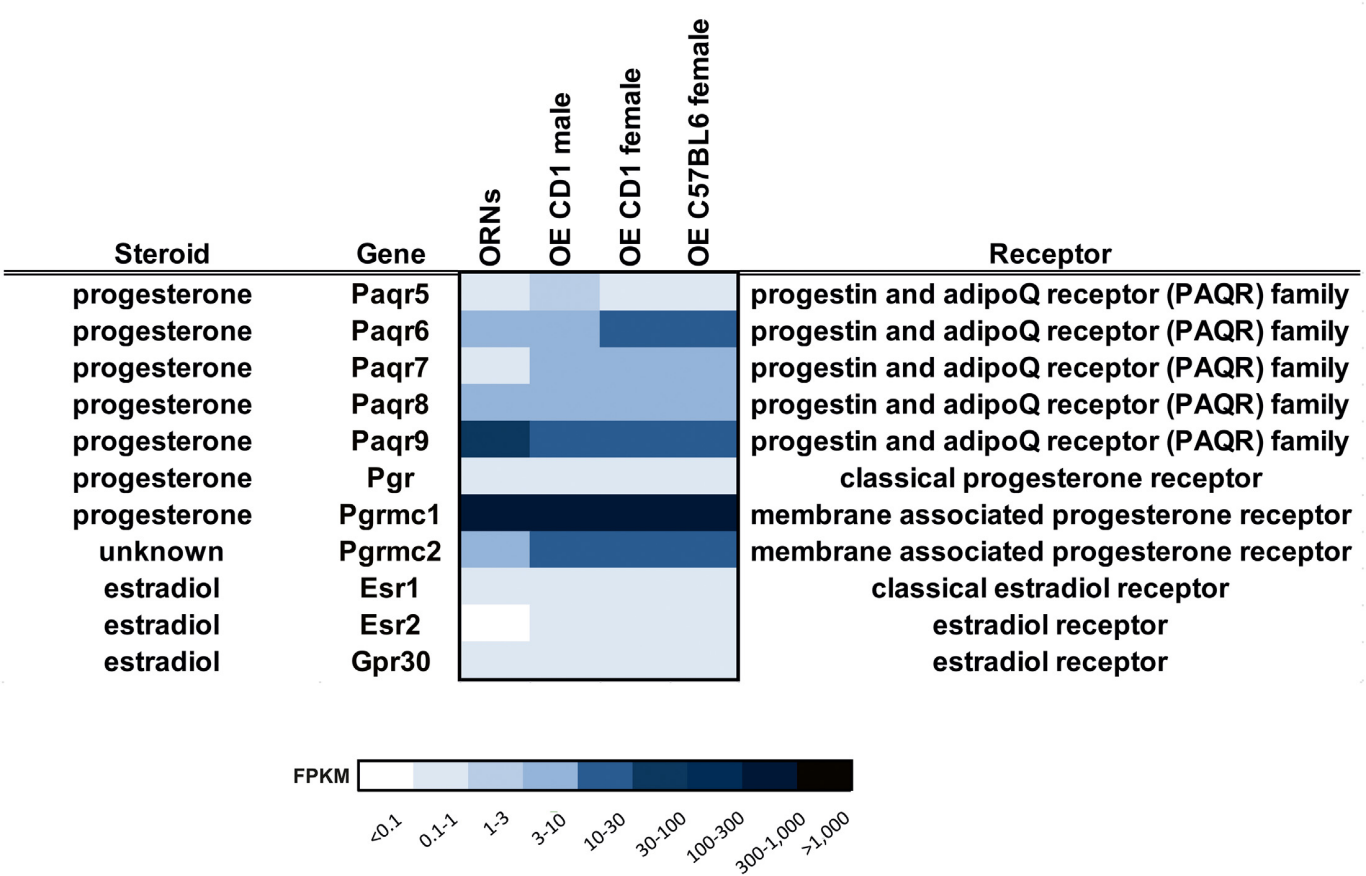
Expression level of steroid receptors detected by RNA-Seq. Heat Map showing the transcript expression level of steroid receptors in ORNs and OE [36]. Deeper colors indicate a higher expression level.

Progesterone can modulate various neuronal functions through different classes of receptors. We screened for the expression of progesterone receptors using RNA-Seq based transcriptome data from the OE and FACS-sorted ORNs [36]. The sequencing data provide a comprehensive overview of the expression levels for the distinct classes of progesterone receptor genes (Fig 1). The classical progesterone receptor (Pgr) was weakly expressed in the OE. Within the PAQR family, which includes the non-classical membrane progesterone receptors, a high mRNA level of Paqr9 was detected (FPKM 30), whereas Paqr6 (FPKM 5) and Paqr8 (FPKM 4) showed lower expression in ORNs. Furthermore, the expression of Pgrmc1 was high in ORNs (FPKM 340).

In addition to progesterone, estradiol is another important female steroid hormone with various functions. Therefore, we screened the RNA-Seq data for possible estrogen receptors (Fig 1). In the ORNs, expression was detected for Esr1 and Gpr30 with FPKM values > 0.6, and Esr2 was not expressed. Additionally, we performed RT-PCR with OE mRNA to verify the RNA-Seq results for several progesterone and estrogen receptors (S1 Fig). To assess sex specific differences in the expression of steroid receptors, we used a cuffdiff analysis as previously described [36]; however, no sex-specific expression patterns were found (S1 Table).

### Localization of progesterone and estradiol receptor proteins in the OE

**Fig 2:**
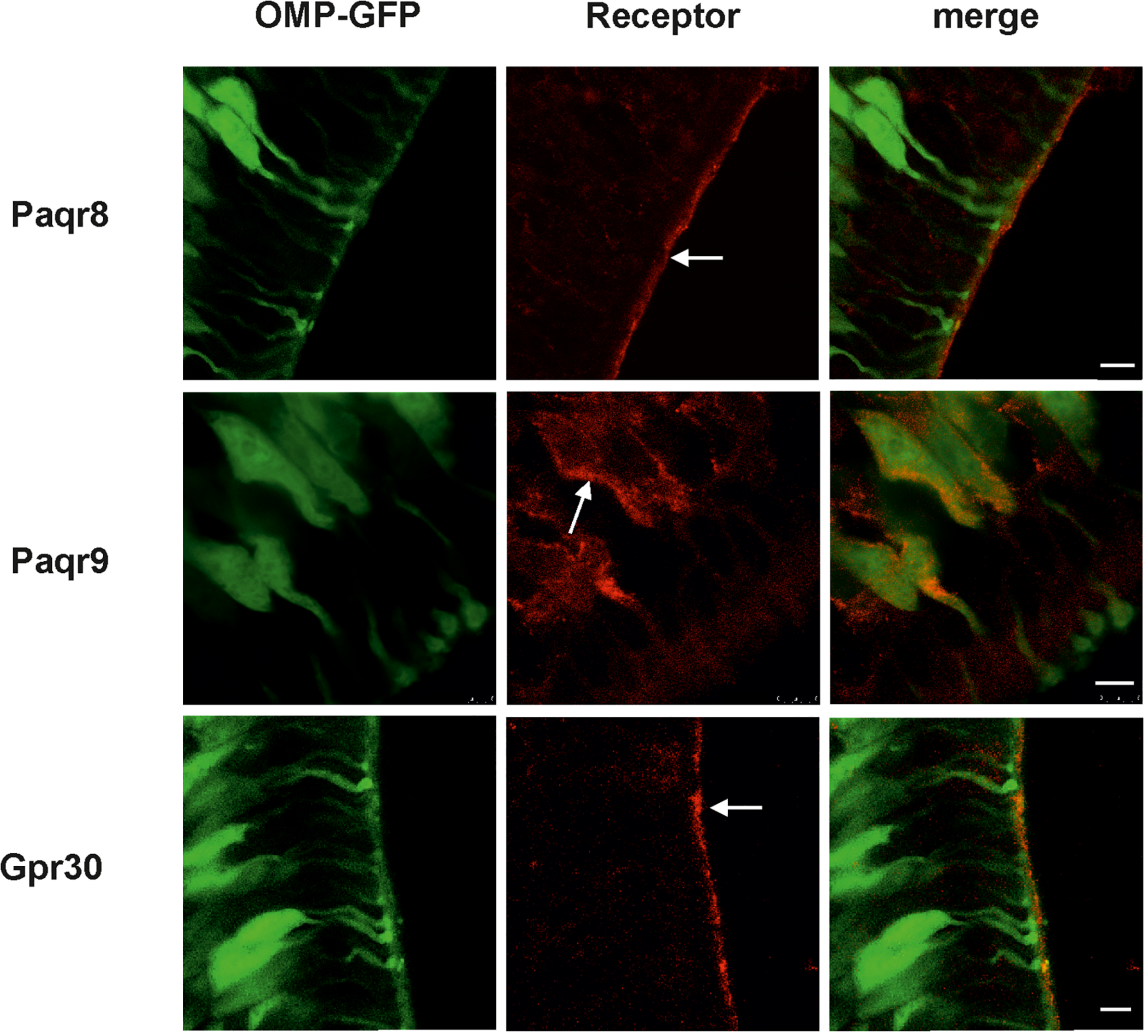
Expression of membrane progestin and estradiol receptors in the OE. Protein expression was assessed by staining coronal sections of OMP-GFP mice with anti-Paqr8, anti-Paqr9 and anti-Gpr30 antibodies. Left: GFP fluorescence (green) served to identify mature ORNs. Center: Paqr9 immunoreactivity (red) was observed in the soma membrane of a subset of mature ORNs, while Paqr8 and Gpr30 immunoreactivity was detected in the cilia membrane as indicated by the arrows. Right: Overlay of the GFP signal and antibody staining. Scale bar, 5 μm. Control staining with the secondary antibody showed low background (S3 Fig).

The subcellular localization of receptor proteins for progesterone and estradiol in the OE, especially in ORNs, remains unknown. Thus, we examined receptor protein expression by immunohistochemistry. The results showed a subpopulation of mature ORNs with immunoreactivity for Paqr9 in the soma membrane and Paqr8 in the cilia membrane of mature ORNs (Fig 2). We also observed a pronounced cilia membrane expression of Gpr30 in the OE. Pgrmc1 could be weakly detected in cilia layer (S2 Fig).

### Progesterone decreased odorant-evoked responses in a dose-dependent manner in the OE

**Fig 3:**
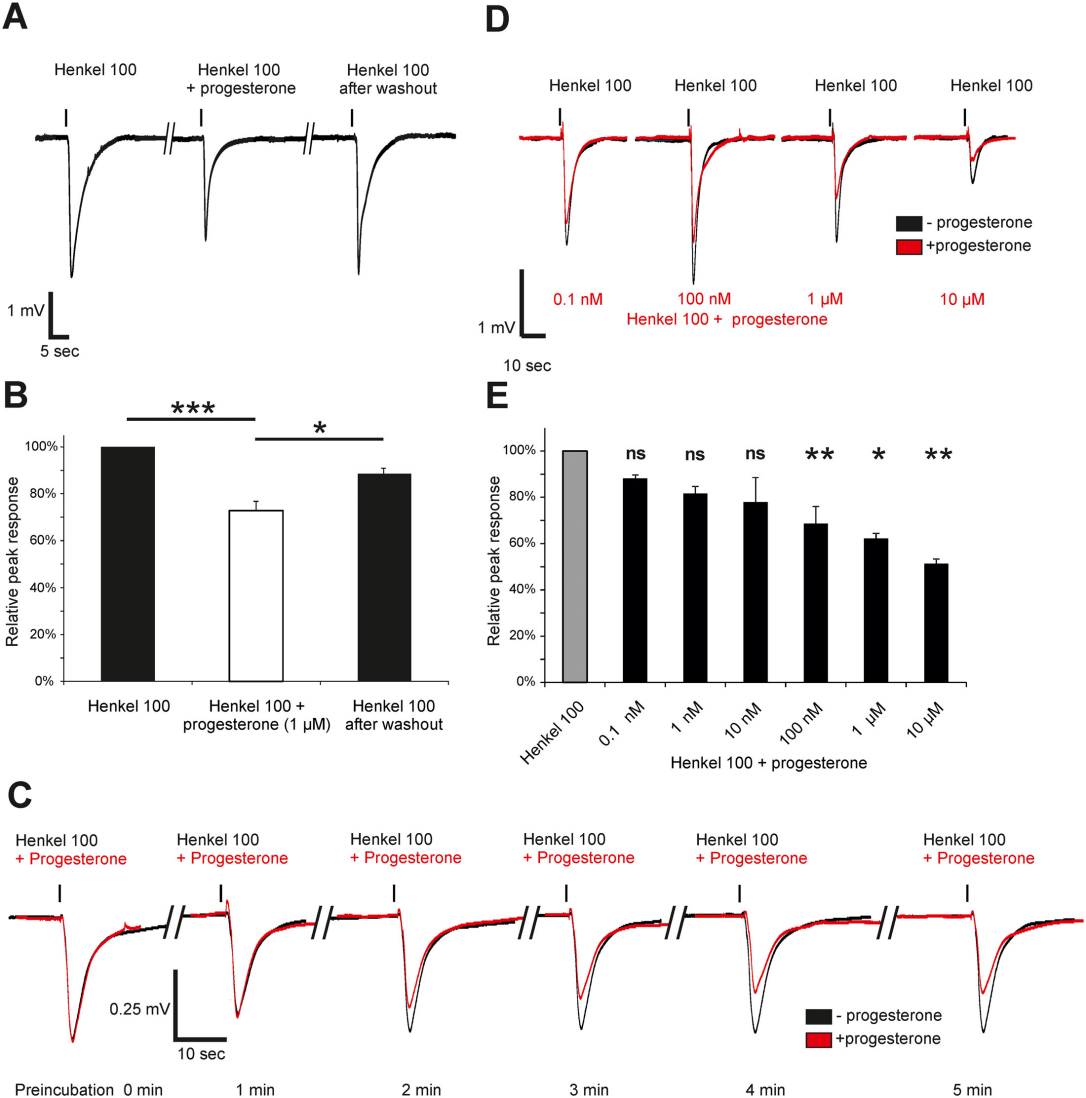
Progesterone decreased Henkel 100-induced responses in a dose-dependent manner in the OE of female mice. (A) Representative submerged EOG esponses to Henkel 100 (1:5,000) were recorded from the surface of the septum from female mouse OE. The first response was recorded as a control, the second response was recorded after a 4-min progesterone preincubation (1 μM) and the third response was recorded after progesterone washout. The amplitude value was decreased after application of progesterone, but recovered after progesterone washout. (B) The graph shows the relative amplitude of the response to Henkel 100, Henkel 100 after progesterone preincubation and after progesterone washout. The response was significantly decreased after preincubation with 1 μM progesterone to 72.8 ± 3.6%, but it recovered to 90.8 ± 2.3% of the starting value after progesterone washout (n = 8). (C) Representative submerged EOG measurements of the responses to Henkel 100 (1:5,000) recorded from the surface of the septum of female mice during (red) and without progesterone (black) incubation. After 2 min of progesterone preincubation, the response decreased. After 3–4 min of preincubation, the response was maximally decreased. (D) Representative submerged EOG responses to Henkel 100 (1:5,000) under control conditions (black) and 4 min after progesterone preincubation at different concentrations (red). (E) Relative reduction in peak response to Henkel 100 after progesterone preincubation at different concentrations (0.1 nM: 11.9 ± 2.1% (n = 3), 1 nM: 18.4 ± 3.1% (n = 3), 10 nM: 22.0 ± 11.4% (n = 4), 100 nM: 31.4 ± 7.3% (n = 4), 1 μM: 37.9 ± 2.2% (n = 4), and 10 μM: 48.8 ± 2.1% (n = 4)). Progesterone reduced the peak response in a dose-dependent manner. Significant data are labeled: *p ≤ 0.05, **p ≤ 0.01 and ***p ≤ 0.001.

Based on the evidence for mPRs in the cilia of ORNs, we hypothesized that progesterone may influence the odorant-evoked responsiveness of neurons. Therefore, we used submerged EOG recordings to measure the electrical activity of a large set of ORNs in the OE of adult female mice. During the recordings, we repetitively stimulated (stimuli interspaced 4 minutes to exclude adaptive processes) the OE with non-saturating concentrations of Henkel 100 as a complex odor mixture (1:5,000) for 500 ms. To determine the effects of progesterone on odorant-evoked responses, we measured the Henkel 100-evoked responses with and without preincubation with progesterone. After incubation with 1 μM progesterone for 4 minutes, we observed a reduced peak amplitude for Henkel 100 that was recovered after progesterone washout (Fig 3A). Progesterone decreased the amplitude of Henkel 100 responses to 72.8% of the control (n = 8, P < 0.01). After wash out, the response recovered significantly (P < 0.01) and the Henkel 100 evoked resonse was restored to 90.8 ± 2.3% of the control (Fig 3B). This effect was dependent on the duration of progesterone preincubation and reached its maximum after 4 minutes (Fig 3C). Prolonged preincubation with progesterone for 8 and 12 minutes did not lead to a larger reduction (data not shown). In control experiments, 1 μM progesterone alone induced no electrical responses in submerged EOGs.

Next, we tested if the modulatory effect was concentration dependent. We were able to record Henkel 100 responses during incubation with six increasing concentrations of progesterone (0.1 nM—10 μM) within the same mouse OE because the inhibitory effect of progesterone on odorant-evoked responses was reversible (Fig 3D and 3E). The odorant-evoked responses were reversibly decreased in a concentration-dependent manner by progesterone. Our submerged EOG recording results revealed that this modulatory effect conveyed through progesterone was significantly present at l00 nM concentrations. At this concentration, the Henkel 100 responses were decreased by 31.4 ± 7.3% (Fig 3D and 3E).

**Fig 4:**
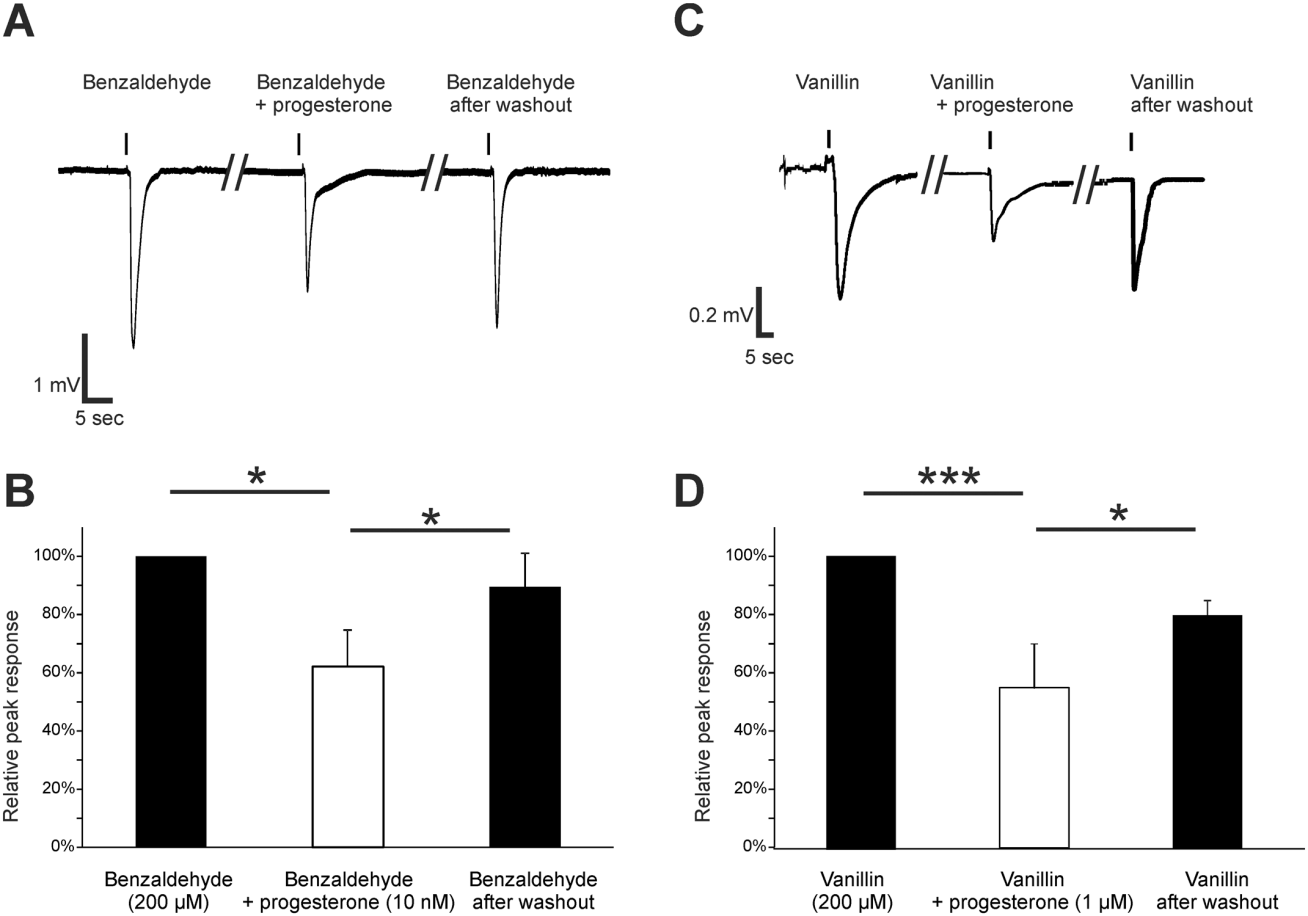
Progesterone decreased benzaldehyde- and vanillin-induced responses in the OE of mice. (A) Representative submerged EOG responses to 200 μM benzaldehyde were recorded from the surface of the septum of male mice. Preincubation with 10 nM progesterone reversibly reduced the benzaldehyde response. (B) The amplitude was significantly decreased after progesterone incubation (37.9 ± 12.3%) and recovered to 89.5 ± 11.6% after progesterone washout (n = 3). (C) Representative submerged EOG responses to 1 mM vanillin were recorded from the surface of the septum of female mice. (D) Preincubation with 1 μM progesterone reversibly reduced the vanillin response. The amplitude was decreased after progesterone incubation (56.0 ± 9.0%) and recovered to 79.4 ± 3.9%) after progesterone washout (n = 4). Significant data are labeled: *p ≤ 0.05, ***p ≤ 0.001.

Furthermore, we showed that the progesterone-mediated effect is not exclusive for complex odorant mixtures. We applied 200 μM benzaldehyde as a stimulus and incubated with 10 nM progesterone. Benzaldehyde responses were reduced (Fig 4A) by 37.9 ± 12.3% and then recovered to 89.5 ± 11.6% after washout (Fig 4B). Additionally, the response evoked by 1 mM vanillin as a further single odorant stimulus was decreased by progesterone to 40.6 ± 2.9% of the control and showed a recovery to 79.8 ± 13.7% after washout (n = 2) (Fig 4C and 4D).

### Progesterone decreased odorant-evoked currents in single ORNs

**Fig 5:**
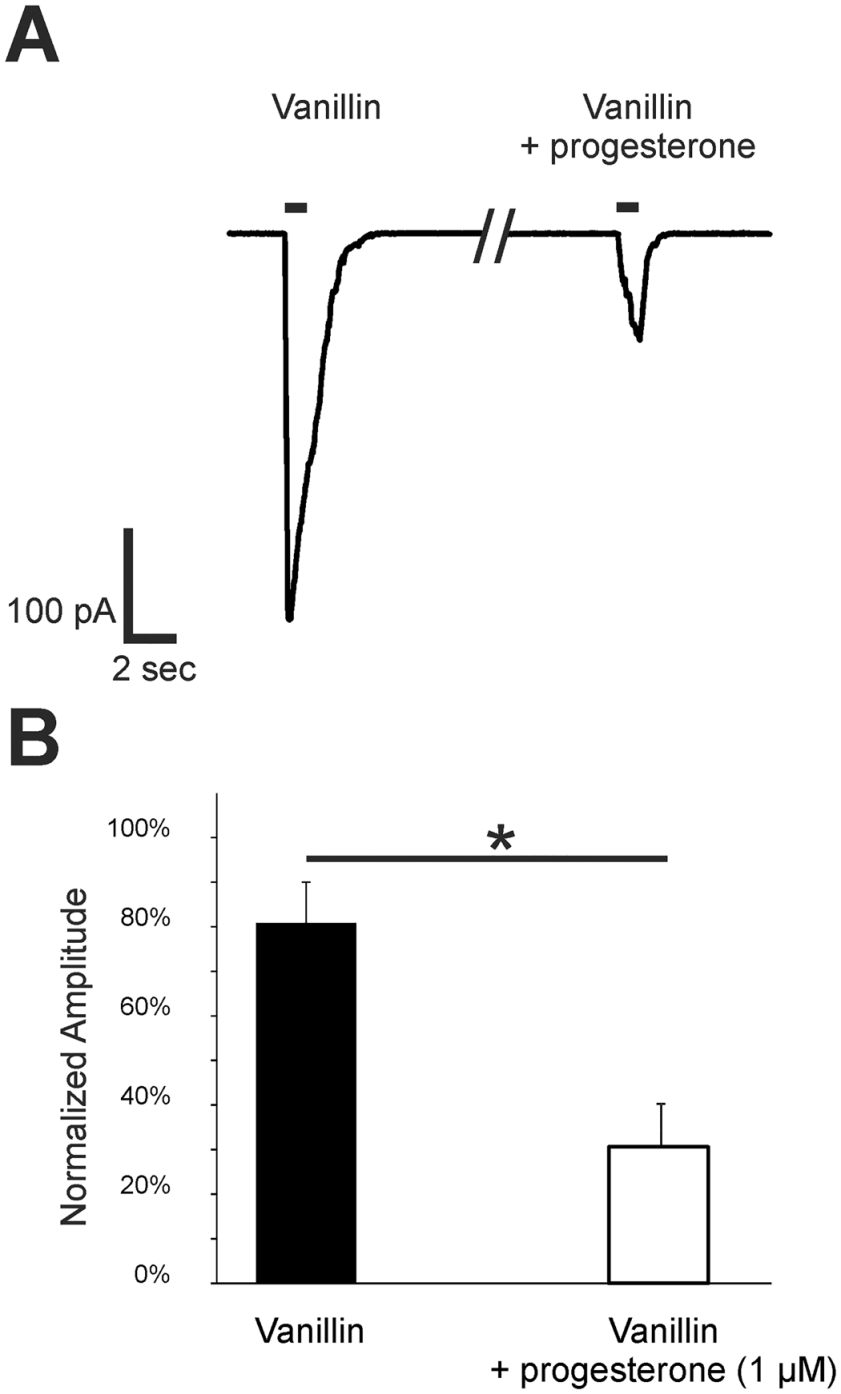
Progesterone decreased vanillin-induced responses in the ORNs of p5 mice. (A) Representative responses to 100 μM vanillin were obtained from Olfr73 positive ORNs in tissue slices using patch-clamp recordings in a voltage-clamp configuration (V_h_ = -56 mV). The amplitude was decreased after a 1-min preincubation with 1 μM progesterone. Application time was 1 s as indicated by the application bar. (B) As a control, neurons were challenged with two subsequent applications of 100 μM vanillin. To characterize the effect of progesterone, 100 μM vanillin was applied followed by a progesterone preincubation and a subsequent second application of vanillin. The bar diagram shows the second response with or without progesterone preincubation relative to the first vanillin application. Relative to the second application of vanillin after 1 min in control neurons, the response of Olfr73 positive neurons was significant decreased in neurons preincubated with 1 μM progesterone for 1 min (n = 7). Significant data are labeled: *p ≤ 0.05.

EOG recordings cannot provide information at the single ORN level; thus, we next investigated the modulatory effect of progesterone on individual ORNs using whole cell voltage-clamp recordings. Here, we used acute OE slices from transgenic mor-EG-GFP mice (P5). This method allows for the recording of Olfr73 (mor-EG-GFP) positive neurons in voltage-clamp mode (V_h_ = -56 mV). For stimulation, we used 100 μM vanillin as an effective ligand for Olfr73 in a nearly saturating concentration [40]. Slices were incubated with 1 μM progesterone. The ORNs showed little to no response to progesterone alone. However, progesterone decreased vanillin-evoked amplitudes after one minute of incubation when compared with the control measurements (Fig 5A). Accordingly, progesterone significantly reduced vanillin-evoked responses of individual ORNs to 31.6 ± 13.1% (n = 7) (Fig 5B). Similar to the EOG data from the OE, the patch-clamp recordings showed a strong modulatory effect of progesterone on ORNs.

### Pharmacological characterization of the endogenous progesterone receptor

**Fig 6:**
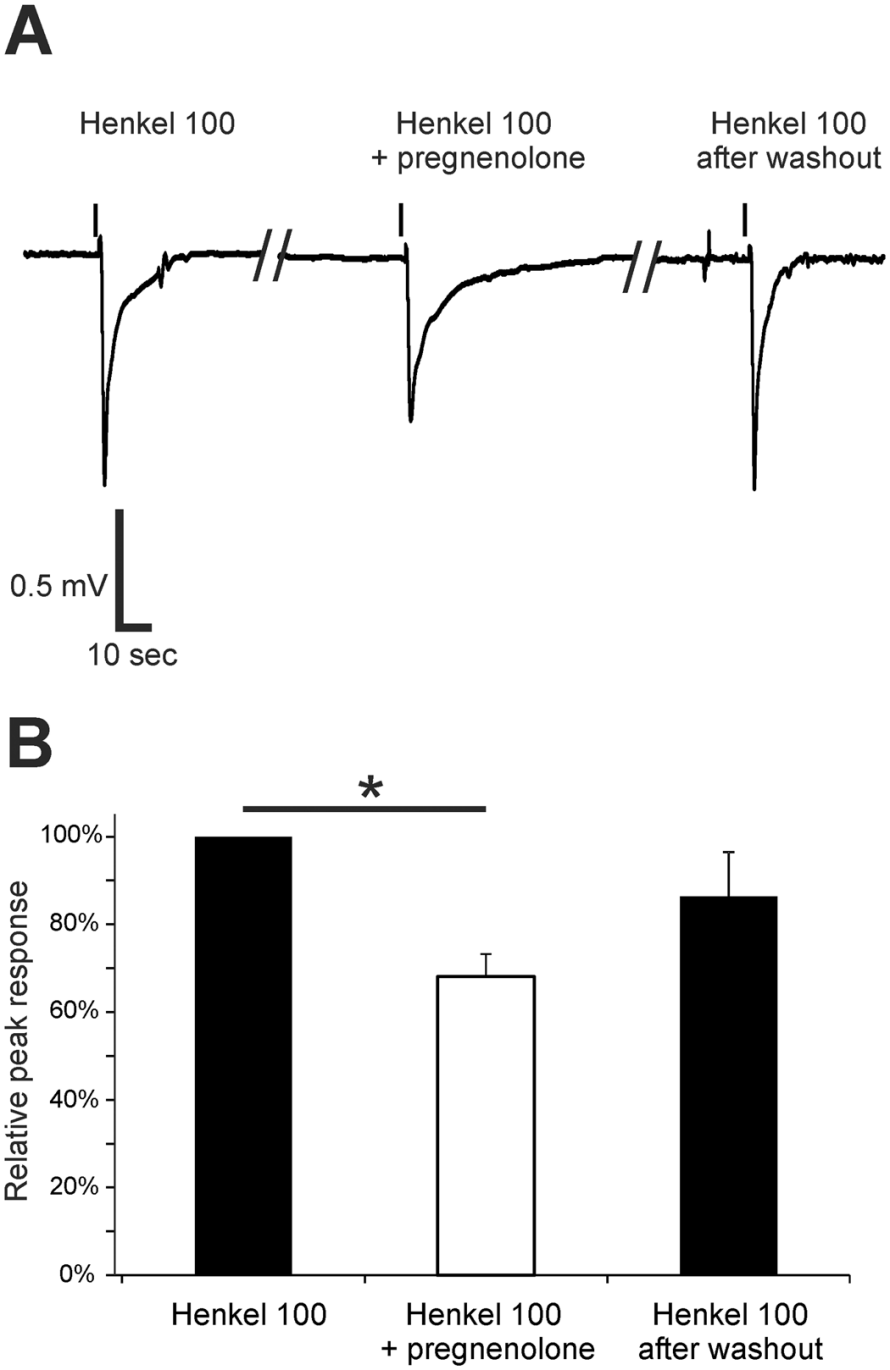
Pregnenolone decreased the effect of Henkel 100-induced responses in the OE. (A) Representative submerged EOG responses to Henkel 100 (1:5,000) were recorded from the surface of the septum of female mice. Pregnenolone preincubation (1 μM) temporarily reduced the response amplitude. (B) The response was significantly decreased after pregnenolone preincubation (31.9 ± 4.8%) and recovered to 86.4 ± 10.2% after washout (n = 3). Significant data are labeled: *p ≤ 0.05.

To further characterize the pharmacological properties of the endogenous receptor in greater detail, we tested substances chemically related to progesterone and known ligands of progestin receptors [41,42]. Pregnenolone, a known agonist of Paqr6 and -9 [24] is the precursor of progesterone, and it is metabolized into progesterone by 3-beta-hydroxysteroid dehydrogenase or delta 4–5 isomerase. We tested pregnenolone at a concentration of 1 μM in submerged EOGs and used Henkel 100 (1:5,000) as a stimulus. We observed a reduced response to the odor mixture during incubation with pregnenolone (Fig 6A). Comparable to the modulatory effect of progesterone, pregnenolone (1 μM) reduced the evoked response by 31.9 ± 8.4% and showed a recovery to 86.4 ± 10.2% of the control after washout (Fig 6B).

**Fig 7:**
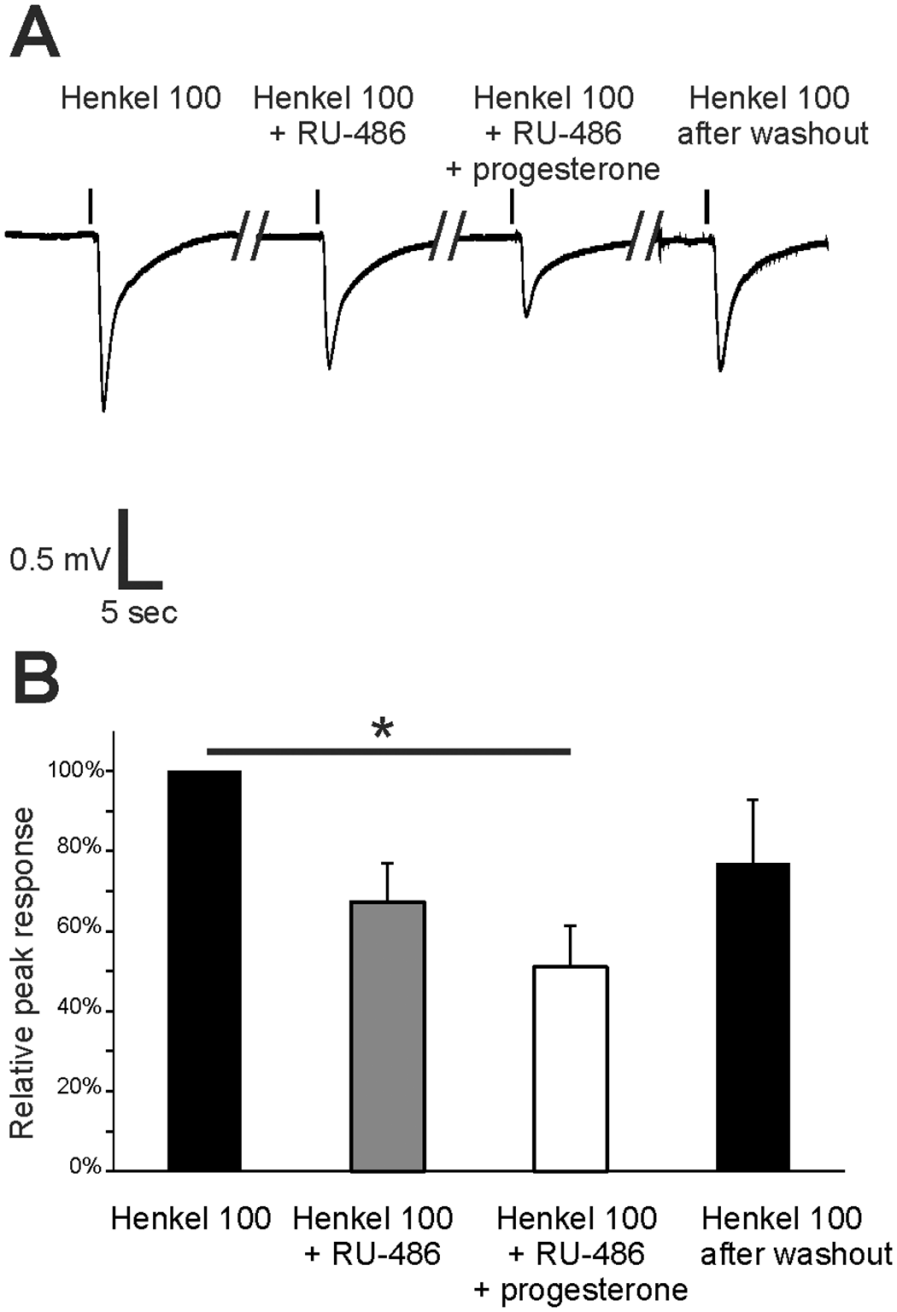
Mifepristone (RU-486) decreased the Henkel 100-induced response in the OE. (A) Representative submerged EOG responses to Henkel 100 (1:5,000) were recorded from the surface of the septum of female mice. The first response was recorded as a control, the second response was recorded after a 4-min RU-486 preincubation (1 μM), the third response was recorded after preincubation with RU-468 and progesterone (1 μM) and the fourth response was recorded after washout. (B) The response to Henkel 100 was decreased after RU-468 preincubation (32.7 ± 9.6%). This effect was further decreased after preincubation with RU-486 and progesterone (49.0 ± 10.4%). The response recovered to 76.9 ± 15.7% after washout (n = 3). Significant data are labeled: *p ≤ 0.05.

Mifepristone (RU-486) acts as a partial antagonist for the classical nuclear progesterone receptor Pgr, but it is an agonist for some mPRs in the low μM range [41]. We determined the effects of 1 μM RU-486 alone and a mixture of RU-486 with 1 μM progesterone on the Henkel 100-evoked response (Fig 7A). After preincubation with RU-486, the amplitude was reduced to 32.7 ± 9.5% when compared with the control. The combination of RU-486 and progesterone further reduced the responses to 49.0 ± 10.4%. This effect was reversible after washout with Ringer`s solution, and the amplitude reached 77.0 ± 15.7% of the starting value (Fig 7B). This result excludes Pgr as a mediator of the modulatory effect because the progesterone effect was not antagonized by RU-486. In contrast, it provides evidence for a role of mPRs because RU-486 possibly acts as an agonist for these receptors [41].

### Estradiol decreased odor-induced responses in the OE and odorant-evoked currents in single ORNs

**Fig 8:**
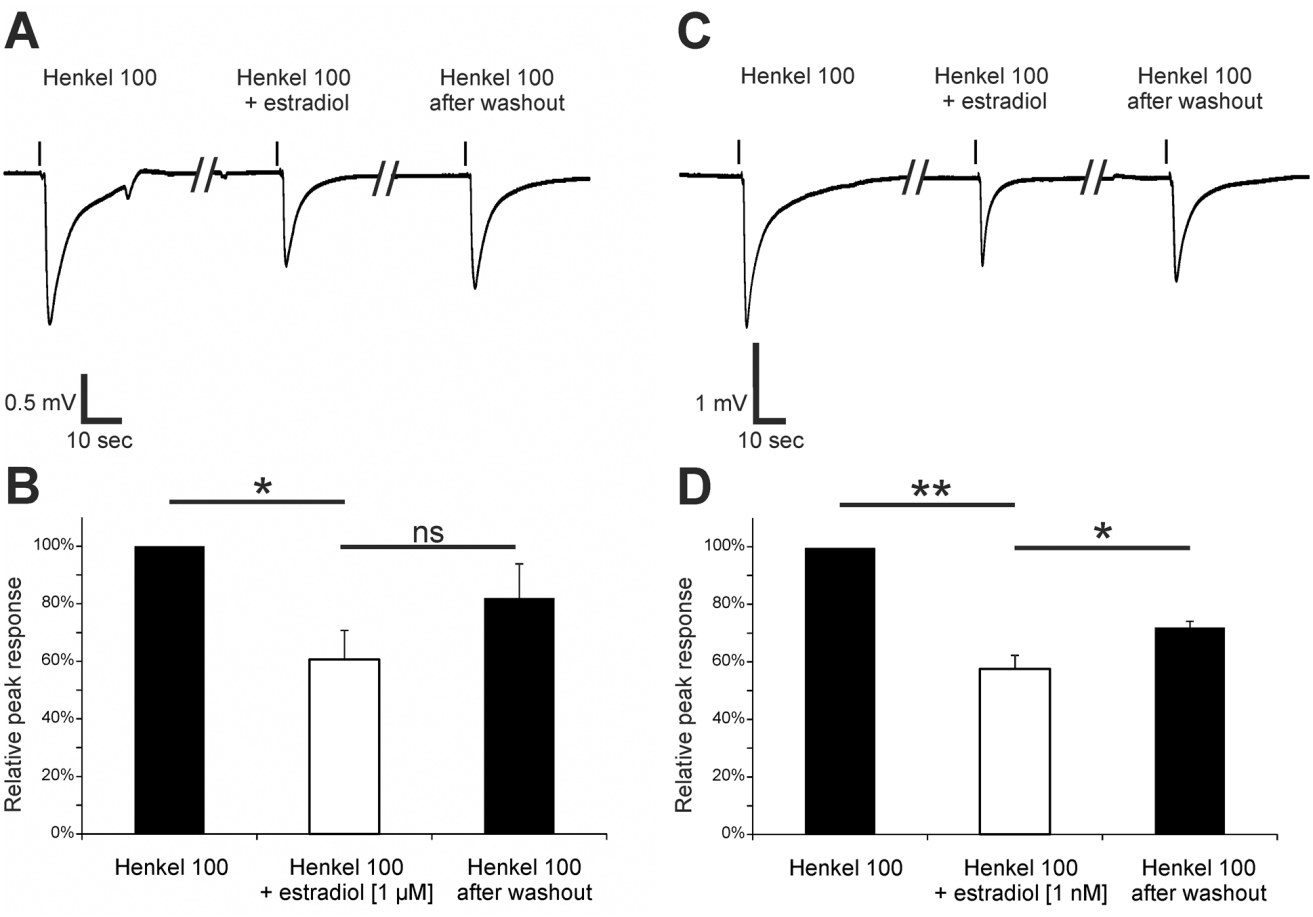
Estradiol affected Henkel 100-induced responses in the OE. (A) Representative submerged EOG responses to Henkel 100 (1:5,000) were recorded from the surface of the septum of female mice. The amplitude was lowered after preincubation with 1 μM estradiol and recovered after washout. (B) The graph shows the relative response amplitudes to Henkel 100, Henkel 100 after estradiol preincubation and after estradiol washout. The response was significantly decreased after estradiol preincubation (39.4 ± 8.3%) and then recovered to 82.0 ± 9.5% after washout (n = 4). (C) Representative submerged EOG responses to Henkel 100 (1:5,000) were recorded under control conditions, after a 4-min preincubation with 1 nM estradiol and after washout. The amplitude value was reduced after preincubation with 1 nM estradiol and recovered after washout. (D) The graph shows the relative response to Henkel 100, Henkel 100 after estradiol preincubation and after estradiol washout. The response was significantly decreased after estradiol preincubation (42.2 ± 3.8%) and recovered to 72.2 ± 2.2% of the starting value after washout (n = 3). Significant data are labeled: *p ≤ 0.05, **p ≤ 0.01.

We demonstrated that proteins of the estradiol receptor Gpr30 are expressed in the cilia of ORNs. Therefore, it is likely that estradiol can rapidly modulate odorant-evoked signals. Submerged EOG recordings demonstrated that application of 1 μM estradiol reduced Henkel 100-evoked signals in the OE (Fig 8A and 8B). Incubation with estradiol significantly decreased the amplitude of the response to Henkel 100 by 39.4 ± 8.3%. The amplitude recovered to 82.0 ± 9.5% of the control after washout (n = 4). Application of 1 nM estradiol also significantly decreased the odorant-evoked responses by 42.2 ± 3.8% with a recovery to 72.2 ± 2.2% of the starting value after washout (n = 3). Thus, estradiol reversibly and strongly decreased odorant-evoked responses at a low nanomolar concentration.

**Fig 9:**
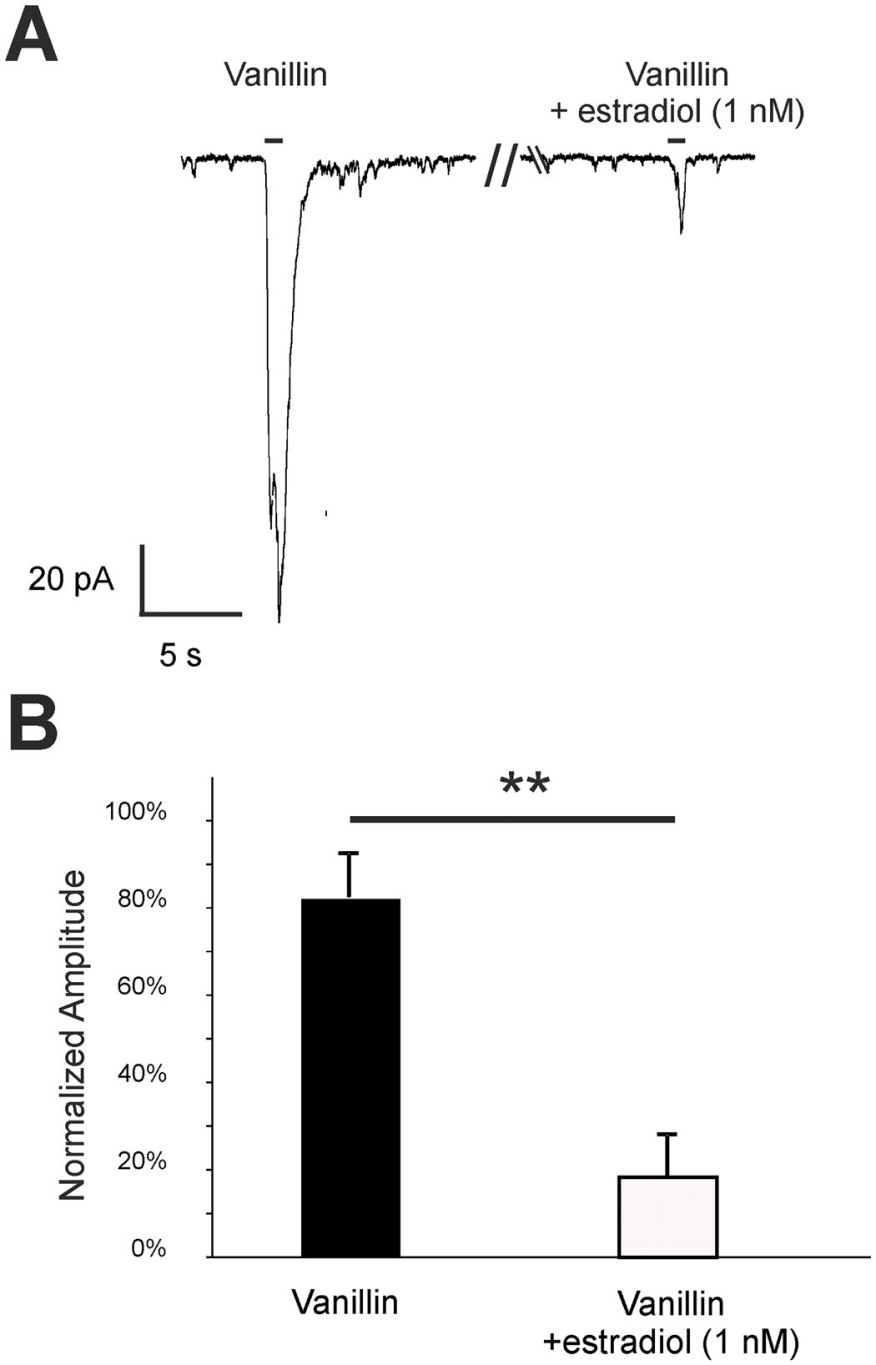
Estradiol decreased vanillin-induced responses in the ORNs of p5 mice. (A) Representative responses to 100 μM vanillin from Olfr73 positive ORNs in tissue slices were obtained from patch-clamp recordings set to a voltage-clamp configuration (V_h_ = -56 mV). The amplitude was decreased after 1-min preincubation with 1 nM estradiol. Application time was 1 s as indicated by the application bar. B) As a control, neurons were challenged with two subsequent applications of 100 μM vanillin. To characterize the effect of progesterone, 100 μM vanillin was applied followed by a estradiol preincubation and a subsequent second application of vanillin. The bar diagram shows the second response with or without estradiol preincubation relative to the first vanillin application. Relative to the second application of vanillin after 1 min in control neurons, the response of Olfr73 positive neurons was significant decreased in neurons preincubated with 1 nM estradiol for 1 min (n = 8). Significant data are labeled: **p ≤ 0.01.

Whole cell voltage-clamp recordings of single Olfr73 positive ORNs showed that estradiol decreased vanillin-evoked responses (V_h_ = -56 mV). After a one minute preincubation with 1 nM estradiol, vanillin-evoked responses of individual ORNs were significantly reduced to 18.0 ± 9.8% (n = 8) (Fig 9). Similar to the EOG data from the OE, patch-clamp recordings showed that estradiol has a strong modulatory effect on odor-evoked responses of single ORNs.

### Progesterone and estradiol decreased odorant-evoked cAMP levels

**Fig 10:**
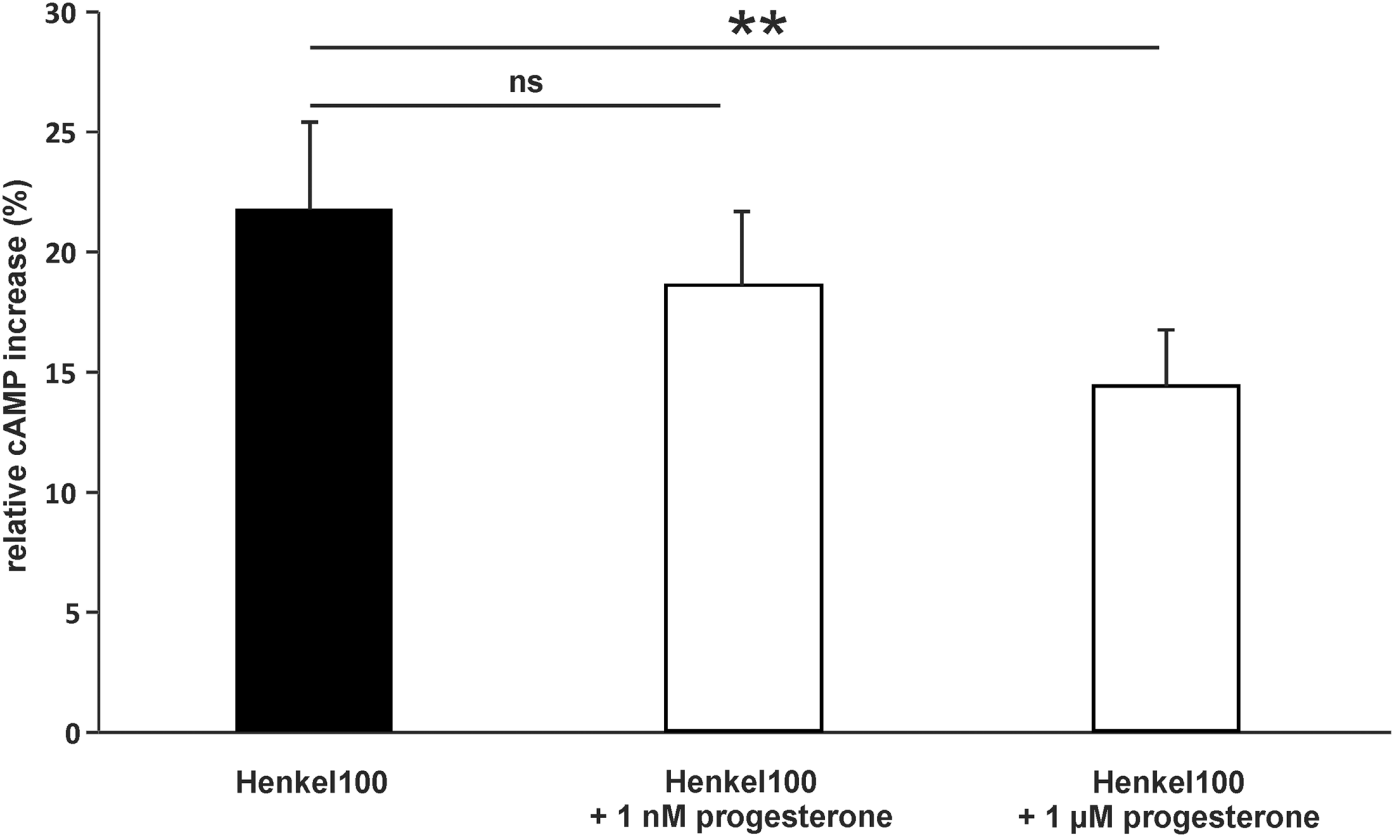
Progesterone decreased odorant-induced cAMP levels in dissociated cells of the OE. Bar diagrams show the percent increase in cAMP compared to control cells after Henkel 100 stimulation (1:100,000). Progesterone preincubation reduced the effect of the Henkel 100 stimulation (n = 4 independent pools of the OE from 10 mice (male and female)). Significant data are labeled: **p ≤ 0.01.

Our results provided strong evidence that progesterone and estradiol modulate odor-evoked responses; thus, we investigated if the rapid effect mediated by these hormones alters odorant-evoked cAMP levels. In dissociated cells of the mouse OE, Henkel 100 stimulation increased the intracellular cAMP level 21.8 ± 3.6% when compared with control cells. After preincubation with 1 μM progesterone, the Henkel 100-induced cAMP increase diminished to 14.3 ± 2.3% relative to the control (Fig 10).

**Fig 11:**
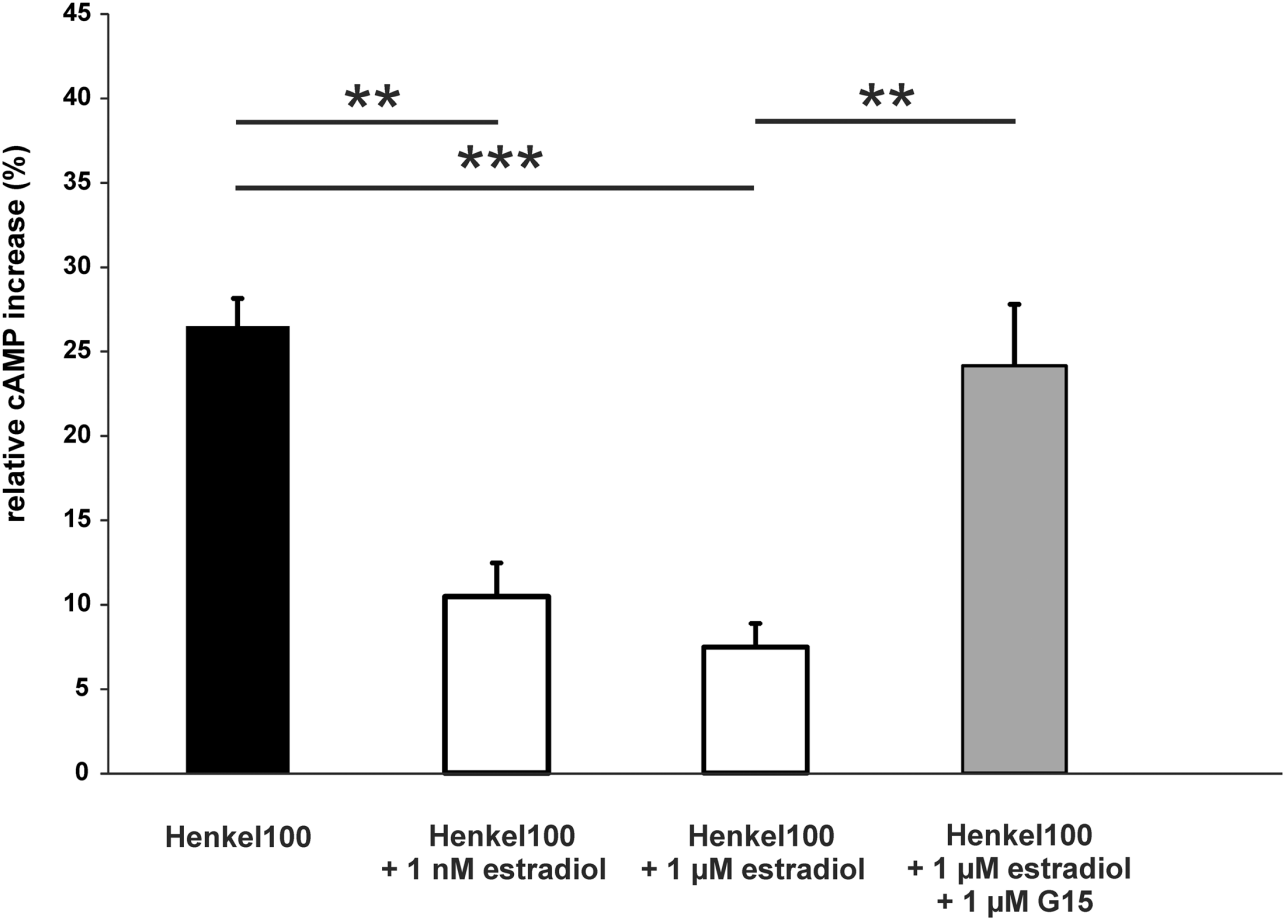
Estradiol decreased odorant-induced cAMP levels. The bar charts represent the relative cAMP levels compared to the negative control in dissociated cells of the OE after Henkel 100 stimulation (1:100,000), Henkel 100 after preincubation with estradiol and Henkel 100 after preincubation with estradiol and the Gpr30-specific antagonist G15 (1 μM, stimulation (n = 3 independent pools of the OE from 10 mice (male and female))). Significant data are labeled: *p ≤ 0.05, **p ≤ 0.01 and ***p ≤ 0.001.

**Fig 12:**
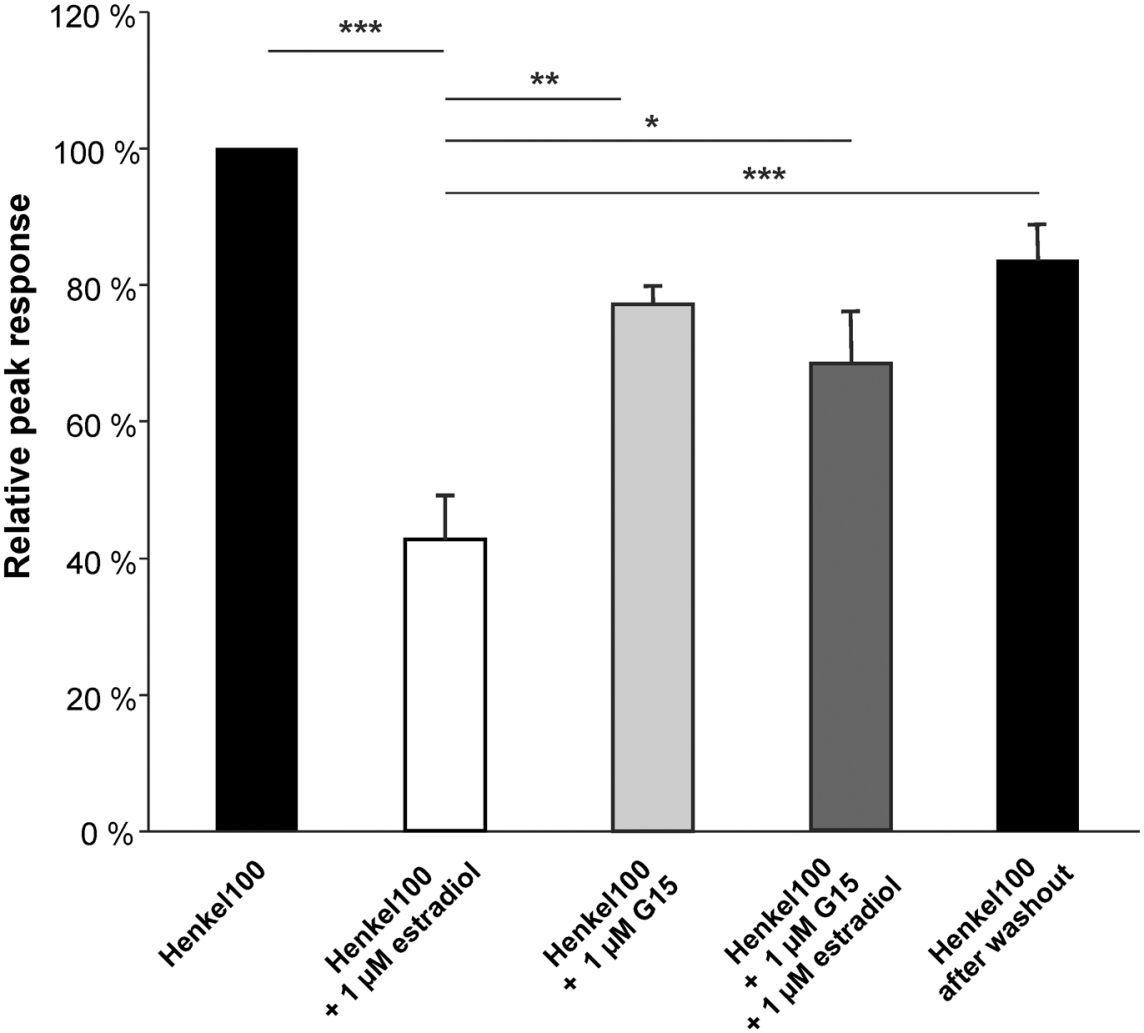
Effect of the Gpr30-specific antagonist G15 on estradiol modulated odorant-evoked responses in submerged EOG recordings from female mice. Bar charts show the relative peak response to Henkel 100 (1:5,000), Henkel 100 after estradiol preincubation, after G15 preincubation, after G15 and estradiol preincubation and after washout. Estradiol preincubation significantly reduced the Henkel 100 response to 42.0 ± 6.1%. The estradiol effect was significantly reduced in the presence of the antagonist G15 (1 μM, 68.9 ± 7.8%). After washout, the response significantly recovered to 83.6 ± 5.3% of control values (n = 6). Significant data are labeled: *p ≤ 0.05, **p ≤ 0.01 and ***p ≤ 0.001.

Also preincubation with 1 nM or 1 μM estradiol significantly decreased odorant-evoked cAMP levels (Fig 11). Application of the Gpr30-specific antagonist G15 abolished the estradiol effect, as shown by cAMP levels similar to the response to Henkel 100 without estradiol. These findings support the idea that estradiol mediates the rapid decrease of odorant-evoked signals via Gpr30. Additionally, results of submerged EOG recordings showed that the antagonist G15 significantly reduced the estradiol mediated decrease of odorant-evoked responses (Fig 12).

## Discussion

Many controversial studies have reported altered olfactory sensitivity via central and peripheral effects that correlates with changes in steroid hormone level in females [8,9,43–48]. Nevertheless, studies focusing on the direct modulation of neurons in the OE are lacking, and the exact molecular mechanisms modulating olfactory perception are unknown.

To the best of our knowledge, we are the first to demonstrate using electrophysiological approaches and cAMP assays that the two sex steroids progesterone and 17-β-estradiol rapidly modulate odorant-evoked signals in mature ORNs. Both of these steroids influence the functional properties of ORNs in mice by significantly decreasing the odorant-evoked responses measured. Both steroids also rapidly modulate these odor responses within minutes. Progesterone and estradiol exert actions at the genomic level via classical nuclear receptors in the time frame of hours [49]. In contrast, non-genomic effects are mediated by hormone-activated GPCRs in the time frame of seconds to minutes due to the activation of second messenger pathways [50] [51].

By screening our NGS-based RNA-Seq transcriptome data of FACS-sorted ORNs for the putative expression of progesterone receptors, we detected the highest expression for Pgrmc1 and the mPR Paqr9. Additional mPRs were expressed at medium (Paqr8 and Paqr6) or low (Paqr5 and Paqr7) levels [36]. No sex-specific differences in transcript levels were found; indeed, we detected similar expression levels for progesterone receptors in male and female OE RNA-Seq transcriptome data [36]. While Paqr9 and Paqr6 activate G_s_, Paqr5, -7, and -8 act through the inhibitory G protein G_i_ [24]. The mPRs are a family of proteins with seven integral TM domains that mediate rapid progestin signaling in diverse model systems. Thus, the ORN-specific expression of these receptors suggests that progesterone exerts its rapid effect through these mPRs. We determined that proteins for Paqr9 are expressed in the soma membrane of ORNs, and Paqr8 expression was strongly concentrated in the cilia layer. This makes both mPRs interesting in the context of a potential modulatory effect on the olfactory signal transduction via activation of G_s_ and G_i_ and their second messenger cAMP pathway.

Progesterone and estradiol can modulate the olfactory transduction cascade; however, the signaling mechanism of both steroids remains elusive. Our experiments do not allow any prediction, if voltage-gated channels were modulated or not. However, the reduced odorant-evoked amplitude in voltage-clamp recordings is independent of a modulation of voltage-gated channels as these are not active at the experimental conditions.

We found decreased levels of cAMP after a preincubation with progesterone, suggesting that progesterone likely targets a protein in the olfactory signaling cascade upstream of ACIII. An altered cAMP level in turn affects the activation of CNG and Cl^-^ channels in the olfactory signaling cascade. Taken together, these results indicate that progesterone reduces receptor potential by inducing a lower cation influx or Cl^-^ efflux due to a reduction in channel activity.

Progesterone reduced odorant-evoked signals in a dose-dependent manner at low nanomolar concentrations. Although studies on local concentrations of progesterone in the OE are lacking, we can estimate the local concentrations from blood plasma levels, which range during estrus and pregnancy from 4.9 ng/ml to 81.9 ng/ml in mice [52]. These values correspond to a concentration range of approximately 15 to 250 nM. We clearly showed an effect at this concentration range; thus, our results suggest that cyclic changes in progesterone can lead to a peripheral modulation of the sensitivity for odor reception. These results lead to the hypothesis that two or more receptors with low to high potency for progesterone are present in ORNs. We found ORN specific expression of Paqr8 and Paqr9 on mRNA and protein level, suggesting that it is probable that both receptors are involved in the progesterone-mediated effect in ORNs. However, both mPRs interact with different G-proteins (Paqr8 with G_i_; Paqr9 with G_s_), and it remains unclear to what extent both receptors contribute to the decreased amplitude response. Additionally, mRNA expression revealed the presence of Paqr6. This protein is coupled to G_s_ and may be a possible receptor mediating the progesterone effect in ORNs. However, the location of this protein in the OE has yet to be determined.

Mifepristone (RU-486), a synthetic steroid with anti-progesterone properties [53], did not antagonize the progesterone-mediated action on odorant-evoked signals. In contrast, RU-486 acted as an agonist comparable to progesterone. RU-486 is an antagonist for classical progesterone receptors [41]; therefore, these receptors can be excluded as progesterone targets. These data highlight the importance of mPR receptors in the OE. Interestingly, Smith et al. showed that RU-486 functioned as a weak agonist for human Paqr7 and Paqr5 expressed in yeast with EC_50_ values in the low μM range [41]. These results indicate that one of the other mPRs likely contributes to the observed affinity to RU-486.

Pang et al. showed that of all of the mPRs expressed in breast cancer cells, only Paqr9 has a high binding affinity for progesterone with an IC_50_ value of 101.6 ± 20.1 nM [24]. This IC_50_ value may correspond to our observed effects of progesterone at higher concentrations. Furthermore, the affinity to the progesterone precursor pregnenolone in our study corresponds to the described binding affinity of Paqr9 to pregnenolone. This affinity is the highest of all tested mPRs [24]. We demonstrated the expression of Hsdb37, an enzyme that converts pregnenolone to progesterone in ORNs. Thus, local production of progesterone may explain the rapid modulation of odor-responses by pregnenolone. These results indicate that several receptors with different binding affinities for progesterone may contribute to the effect on the responsiveness of ORNs to odors.

Recently, it was reported that progesterone leads to a selective blockade of a subpopulation of sensory neurons in the VNO. Pgrcm1 was vital for the progesterone effect as demonstrated by KO-mice, and the downstream phosphorylation of PLCß2 was necessary [4]. The high expression of Pgrmc1 in ORNs suggests a similar role for progesterone modulation in the ORNs. However, whether Pgrmc1 binds progesterone and is itself a progesterone receptor is still a matter of controversy [54,55]. Several studies indicate that Pgrmc1 is part of a multi-steroid binding complex [56,57,54], and other receptor components associated with Pgrmc1 in the VNO or OE have yet to be identified. However, the high expression of Pgrmc1 suggests that it could be part of a multi-protein complex that is important for the action of progesterone in the OE.

In our study, we showed that estradiol rapidly decreased odorant-evoked responses. Low concentrations of 1 nM estradiol exerted a strong effect. Thus, the putative receptor for estradiol seems to be highly sensitive. The low activating concentration of estradiol used in our study is in accordance with the plasma concentrations of estradiol that range from 5.6 pg/ml to 59.7 pg/ml (approximately 0.02–0.2 nM) in the blood of rodents during estrus [58].

The GPCR Gpr30, also named GPER30, is known to mediate the rapid non-genomic effects of estradiol [59]. Immunoreactivity for Gpr30 in the cilia of ORNs indicates that this receptor mediates the rapid action of estradiol in ORNs. Moreover, Gpr30 contains calmodulin-binding domains [60], which suggests a participation in olfactory adaptation similar to that of progesterone. RNA-Seq data revealed the gene expression of aromatase (Cyp19a1) in ORNs, an enzyme necessary for estradiol synthesis.

The presence of this enzyme in ORNs may contribute to the physiological effect of estradiol in ORNs. Therefore, the already observed enhanced urinary odor discrimination in female aromatase knockout (ArKO) mice [61] could be explained by a decrease of the estradiol effect due to a lower estradiol concentrations. Recent studies demonstrated that estradiol and progesterone mediate rapid modulatory effects on mouse vomeronasal sensory neurons by decreasing pheromone responses [62][4]. This effect supports our observations in sensory neurons of the OE. We have provided evidence that the estradiol effect on odorant-evoked responses is likely mediated via the receptor Gpr30 because the Gpr30-specific antagonist G15 diminished the rapid estradiol effect.

Our study provides strong evidence that smell sensitivity is modulated by progesterone and estradiol at the peripheral level by reducing the responsiveness of ORNs. Thus, olfactory sense and endocrine status are linked modalities that mutually influence each other. Reproductively active individuals need facilitation to respond to chemosensory cues and thus maximize or minimize mating and reproduction. For example, olfactory preferences in females change during the estrous cycle. Female rodents show when they are most fertile and exhibit the greatest discrimination between sexual potent and impotent males [63]. In humans, female changes in olfactory preference to male odors correlate with phases of fertility during their menstrual cycle [11,46,45,64]. The enhancement and attenuation of olfactory sensitivity according to the circulating concentrations of steroid hormones may trigger appropriate behavioral and neuronal mechanisms in reproductively active and inactive individuals. In the VNO, progesterone modulates a subset of chemosensory cues [4]. Whether the modulation of sensitivity in the OE is also specific for a subset of possibly sex-related odors is currently unknown.

To our knowledge, we are the first to demonstrate that progesterone and estradiol rapidly act on the ORNs by decreasing odor responses; in addition, these steroids act at the peripheral level in a similar manner as reported for the VNO. Membrane receptors for progesterone and estradiol are coupled to G proteins G_s_/G_i_ and are present in ORNs. Subsequently, both steroids might act via these receptors. Future studies are needed to identify which of the progesterone and estradiol receptors are responsible for the ORN effect. Knockout mice would advance studies on receptor-steroid interactions.

## Supporting Information

S1 Fig RT-PCR analysis of GPCRs obtained from RNA-Seq. Gel electrophoresis of the amplified PCR products from cDNA samples of female murine OE. Used primer pairs were intron-spanning excluding gDNA amplified products.
(DOCX)

S2 Fig Expression of membrane progestin receptors in the OE. Protein expression was assessed by staining coronal sections of OMP-GFP mice with anti-Pgrmc1. Left: GFP fluorescence (green) served to identify mature ORNs. Center: Pgrmc1 immunoreactivity (red) was observed in the cilia layer as indicated by white arrows. Right: Overlay of the GFP signal and antibody staining. Scale bar, 5 μm.
(DOCX)

S3 Fig Control staining of coronal sections of OMP-GFP mice with the secondary antibody. Left: GFP fluorescence (green) served to identify mature ORNs. Center: Only a low background staining is observed using the secondary antibody. Right: Overlay of the GFP signal and antibody staining. Scale bar, 5 μm.
(DOCX)

S1 Table Sex specific gene expression. To assess sex specific differences in the expression of steroid receptors in female (fOE) and male OE (mOE), we used a cuffdiff analysis as previously described [36]; however, no sex-specific expression patterns were found.
(DOCX)

## References

[1] Loch D, Heidel C, Breer H, Strotmann J (2013) Adiponectin enhances the responsiveness of the olfactory system. PloS one 8 (10): e75716 10.1371/journal.pone.0075716 24130737 PMC3794965

[2] Negroni J, Meunier N, Monnerie R, Salesse R, Baly C, Caillol M et al (2012) Neuropeptide Y enhances olfactory mucosa responses to odorant in hungry rats. PloS one 7 (9): e45266 23024812 10.1371/journal.pone.0045266 PMC3443224

[3] Savigner A, Duchamp-Viret P, Grosmaitre X, Chaput M, Garcia S, Ma M et al (2009) Modulation of spontaneous and odorant-evoked activity of rat olfactory sensory neurons by two anorectic peptides, insulin and leptin. Journal of neurophysiology 101 (6): 2898–2906. 10.1152/jn.91169.2008 19297511 PMC2694118

[4] Dey S, Chamero P, Pru JK, Chien M, Ibarra-Soria X, Spencer KR et al (2015) Cyclic Regulation of Sensory Perception by a Female Hormone Alters Behavior. Cell 161 (6): 1334–1344. 10.1016/j.cell.2015.04.052 26046438 PMC4501503

[5] Coiret G, Matifat F, Hague F, Ouadid-Ahidouch H (2005) 17-beta-estradiol activates maxi-K channels through a non-genomic pathway in human breast cancer cells. FEBS letters 579 (14): 2995–3000. 15893312 10.1016/j.febslet.2005.02.085

[6] Lee DY, Chai YG, Lee EB, Kim KW, Nah S, Oh TH et al (2002) 17Beta-estradiol inhibits high-voltage-activated calcium channel currents in rat sensory neurons via a non-genomic mechanism. Life sciences 70 (17): 2047–2059. 12148697 10.1016/s0024-3205(01)01534-x

[7] Vicini E, Loiarro M, Di Agostino S, Corallini S, Capolunghi F, Carsetti R et al (2006) 17-beta-estradiol elicits genomic and non-genomic responses in mouse male germ cells. Journal of cellular physiology 206 (1): 238–245. 15991248 10.1002/jcp.20454

[8] Kumar KR, Archunan G (1999) Influence of the stage of the cycle on olfactory sensitivity in laboratory mice. Indian journal of experimental biology 37 (3): 317–318. 10641165

[9] Pietras RJ, Moulton DG (1974) Hormonal influences on odor detection in rats: changes associated with the estrous cycle, pseudopregnancy, ovariectomy, and administration of testosterone propionate. Physiology & behavior 12 (3): 475–491. 4820142 10.1016/0031-9384(74)90125-5

[10] Numan M, Smith HG (1984) Maternal behavior in rats: evidence for the involvement of preoptic projections to the ventral tegmental area. Behavioral neuroscience 98 (4): 712–727. 6087844 10.1037//0735-7044.98.4.712

[11] Gangestad SW, Thornhill R (1998) Menstrual cycle variation in women's preferences for the scent of symmetrical men. Proceedings. Biological sciences / The Royal Society 265 (1399): 927–933. 9633114 10.1098/rspb.1998.0380 PMC1689051

[12] Gilbert AN, Wysocki CJ (1991) Quantitative assessment of olfactory experience during pregnancy. Psychosomatic medicine 53 (6): 693–700. 1758952 10.1097/00006842-199111000-00009

[13] Shughrue PJ, Lane MV, Merchenthaler I (1997) Comparative distribution of estrogen receptor-alpha and -beta mRNA in the rat central nervous system. The Journal of comparative neurology 388 (4): 507–525. 9388012 10.1002/(sici)1096-9861(19971201)388:4<507::aid-cne1>3.0.co;2-6

[14] Guo XZ, Su JD, Sun QW, Jiao BH (2001) Expression of estrogen receptor (ER) -alpha and -beta transcripts in the neonatal and adult rat cerebral cortex, cerebellum, and olfactory bulb. Cell research 11 (4): 321–324. 11787778 10.1038/sj.cr.7290103

[15] Horvath TL, Wikler KC (1999) Aromatase in developing sensory systems of the rat brain. Journal of neuroendocrinology 11 (2): 77–84. 10048462 10.1046/j.1365-2826.1999.00285.x

[16] Pierman S, Sica M, Allieri F, Viglietti-Panzica C, Panzica GC, Bakker J (2008) Activational effects of estradiol and dihydrotestosterone on social recognition and the arginine-vasopressin immunoreactive system in male mice lacking a functional aromatase gene. Hormones and behavior 54 (1): 98–106. 10.1016/j.yhbeh.2008.02.001 18346740 PMC2706693

[17] Pierman S, Tirelli E, Douhard Q, Baum MJ, Bakker J (2006) Male aromatase knockout mice acquire a conditioned place preference for cocaine but not for contact with an estrous female. Behavioural brain research 174 (1): 64–69. 16942806 10.1016/j.bbr.2006.07.002

[18] Maruska KP, Fernald RD (2010) Reproductive status regulates expression of sex steroid and GnRH receptors in the olfactory bulb. Behavioural brain research 213 (2): 208–217. 10.1016/j.bbr.2010.04.058 20466023 PMC2902620

[19] Sanchez-Andrade G, Kendrick KM (2009) The main olfactory system and social learning in mammals. Behavioural brain research 200 (2): 323–335. 10.1016/j.bbr.2008.12.021 19150375

[20] Nilsen J, Brinton RD (2003) Divergent impact of progesterone and medroxyprogesterone acetate (Provera) on nuclear mitogen-activated protein kinase signaling. Proceedings of the National Academy of Sciences of the United States of America 100 (18): 10506–10511. 12925744 10.1073/pnas.1334098100 PMC193591

[21] Baudry M, Bi X, Aguirre C (2013) Progesterone-estrogen interactions in synaptic plasticity and neuroprotection. Neuroscience 239: 280–294. 10.1016/j.neuroscience.2012.10.051 23142339 PMC3628409

[22] Bali N, Arimoto JM, Iwata N, Lin SW, Zhao L, Brinton RD et al (2012) Differential responses of progesterone receptor membrane component-1 (Pgrmc1) and the classical progesterone receptor (Pgr) to 17β-estradiol and progesterone in hippocampal subregions that support synaptic remodeling and neurogenesis. Endocrinology 153 (2): 759–769. 10.1210/en.2011-1699 22147012 PMC3275384

[23] ter Horst J P, de Kloet E R, Schächinger H, Oitzl MS (2012) Relevance of stress and female sex hormones for emotion and cognition. Cellular and molecular neurobiology 32 (5): 725–735. 10.1007/s10571-011-9774-2 22113371 PMC3377901

[24] Pang Y, Dong J, Thomas P (2013) Characterization, neurosteroid binding and brain distribution of human membrane progesterone receptors δ and {epsilon} (mPRδ and mPR{epsilon}) and mPRδ involvement in neurosteroid inhibition of apoptosis. Endocrinology 154 (1): 283–295. 10.1210/en.2012-1772 23161870 PMC3529379

[25] Petersen SL, Intlekofer KA, Moura-Conlon PJ, Brewer DN, Del Pino Sans J, Lopez JA (2013) Nonclassical progesterone signalling molecules in the nervous system. Journal of neuroendocrinology 25 (11): 991–1001. 10.1111/jne.12060 23763432

[26] Petersen SL, Intlekofer KA, Moura-Conlon PJ, Brewer DN, Del Pino Sans Javier, Lopez JA (2013) Novel progesterone receptors: neural localization and possible functions. Frontiers in neuroscience 7: 164 10.3389/fnins.2013.00164 24065878 PMC3776953

[27] Thomas P (2008) Characteristics of membrane progestin receptor alpha (mPRalpha) and progesterone membrane receptor component 1 (PGMRC1) and their roles in mediating rapid progestin actions. Frontiers in neuroendocrinology 29 (2): 292–312. 10.1016/j.yfrne.2008.01.001 18343488 PMC2600886

[28] Chen J, Lin DJ, Liu MS, Chien EJ (2014) Non-genomic rapid responses via progesterone in human peripheral T cells are not indirectly mimicked by sphingosine 1-phosphate. Steroids 81: 9–12. 10.1016/j.steroids.2013.11.011 24269742

[29] Morrill GA, Kostellow AB, Gupta RK (2013) A computational analysis of non-genomic plasma membrane progestin binding proteins: signaling through ion channel-linked cell surface receptors. Steroids 78 (12–13): 1233–1244. 10.1016/j.steroids.2013.08.006 24012561

[30] Moussatche P, Lyons TJ (2012) Non-genomic progesterone signalling and its non-canonical receptor. Biochemical Society transactions 40 (1): 200–204. 10.1042/BST20110638 22260690

[31] Peluso JJ, Liu X, Gawkowska A, Johnston-MacAnanny E (2009) Progesterone activates a progesterone receptor membrane component 1-dependent mechanism that promotes human granulosa/luteal cell survival but not progesterone secretion. The Journal of clinical endocrinology and metabolism 94 (7): 2644–2649. 10.1210/jc.2009-0147 19417032 PMC2708946

[32] Tang YT, Hu T, Arterburn M, Boyle B, Bright JM, Emtage PC et al (2005) PAQR proteins: a novel membrane receptor family defined by an ancient 7-transmembrane pass motif. Journal of molecular evolution 61 (3): 372–380. 16044242 10.1007/s00239-004-0375-2

[33] Tubbs C, Thomas P (2009) Progestin signaling through an olfactory G protein and membrane progestin receptor-alpha in Atlantic croaker sperm: potential role in induction of sperm hypermotility. Endocrinology 150 (1): 473–484. 10.1210/en.2008-0512 18801904

[34] Thomas P, Dressing G, Pang Y, Berg H, Tubbs C, Benninghoff A et al (2006) Progestin, estrogen and androgen G-protein coupled receptors in fish gonads. Steroids 71 (4): 310–316. 16289637 10.1016/j.steroids.2005.09.015

[35] Thomas P, Pang Y, Dong J, Groenen P, Kelder J, de Vlieg J et al (2007) Steroid and G protein binding characteristics of the seatrout and human progestin membrane receptor alpha subtypes and their evolutionary origins. Endocrinology 148 (2): 705–718. 17082257 10.1210/en.2006-0974

[36] Kanageswaran N, Demond M, Nagel M, Schreiner, Benjamin S P, Baumgart S, Scholz P et al (2015) Deep sequencing of the murine olfactory receptor neuron transcriptome. PloS one 10 (1): e0113170 10.1371/journal.pone.0113170 25590618 PMC4295871

[37] Potter SM, Zheng C, Koos DS, Feinstein P, Fraser SE, Mombaerts P (2001) Structure and emergence of specific olfactory glomeruli in the mouse. The Journal of neuroscience: the official journal of the Society for Neuroscience 21 (24): 9713–9723. 11739580 10.1523/JNEUROSCI.21-24-09713.2001 PMC2570017

[38] Oka Y, Nakamura A, Watanabe H, Touhara K (2004) An odorant derivative as an antagonist for an olfactory receptor. Chemical senses 29 (9): 815–822. 15574817 10.1093/chemse/bjh247

[39] Spehr M, Kelliher KR, Li X, Boehm T, Leinders-Zufall T, Zufall F (2006) Essential role of the main olfactory system in social recognition of major histocompatibility complex peptide ligands. The Journal of neuroscience: the official journal of the Society for Neuroscience 26 (7): 1961–1970. 16481428 10.1523/JNEUROSCI.4939-05.2006 PMC6674934

[40] Baumgart S, Jansen F, Bintig W, Kalbe B, Herrmann C, Klumpers F et al (2014) The scaffold protein MUPP1 regulates odorant-mediated signaling in olfactory sensory neurons. Journal of cell science 127 (Pt 11): 2518–2527. 24652834 10.1242/jcs.144220

[41] Smith JL, Kupchak BR, Garitaonandia I, Hoang LK, Maina AS, Regalla LM et al (2008) Heterologous expression of human mPRalpha, mPRbeta and mPRgamma in yeast confirms their ability to function as membrane progesterone receptors. Steroids 73 (11): 1160–1173. 10.1016/j.steroids.2008.05.003 18603275 PMC2597464

[42] Thomas P, Pang Y (2012) Membrane progesterone receptors: evidence for neuroprotective, neurosteroid signaling and neuroendocrine functions in neuronal cells. Neuroendocrinology 96 (2): 162–171. 10.1159/000339822 22687885 PMC3489003

[43] Eliasson M, Meyerson BJ (1975) Sexual preference in female rats during estrous cycle, pregnancy and lactation. Physiology & behavior 14 (6): 705–710. 1237902 10.1016/0031-9384(75)90061-x

[44] Albertazzi P (2002) Effects of progestins on olfactory sensitivity and cognition. Climacteric: the journal of the International Menopause Society 5 (3): 302; author reply 302–3. 12422897

[45] Doty RL, Cameron EL (2009) Sex differences and reproductive hormone influences on human odor perception. Physiology & behavior 97 (2): 213–228. 19272398 10.1016/j.physbeh.2009.02.032 PMC2693767

[46] Doty RL, Kisat M, Tourbier I (2008) Estrogen replacement therapy induces functional asymmetry on an odor memory/discrimination test. Brain research 1214: 35–39. 10.1016/j.brainres.2008.04.017 18466883 PMC2481562

[47] Hughes LF, McAsey ME, Donathan CL, Smith T, Coney P, Struble RG (2002) Effects of hormone replacement therapy on olfactory sensitivity: cross-sectional and longitudinal studies. Climacteric: the journal of the International Menopause Society 5 (2): 140–150. 12051109

[48] Nathan BP, Tonsor M, Struble RG (2012) Long-term effects of estradiol replacement in the olfactory system. Experimental neurology 237 (1): 1–7. 10.1016/j.expneurol.2012.06.001 22691461 PMC3418380

[49] Guerriero G, Roselli CE, Ciarcia G (2009) The amphibian (Rana esculenta) brain progesterone receptor: relationship to plasma steroids and vitellogenic cycle during the gonadal recovery phase. Annals of the New York Academy of Sciences 1163: 407–409. 10.1111/j.1749-6632.2009.04438.x 19456372

[50] Hammes SR, Levin ER (2011) Minireview: Recent advances in extranuclear steroid receptor actions. Endocrinology 152 (12): 4489–4495. 10.1210/en.2011-1470 22028449 PMC3858720

[51] Bi G, Chen YZ (1999) [The rapid effects of steroids on glycine uptake in neuroblastoma cell strain SK-N-SH cells]. Sheng li xue bao: [Acta physiologica Sinica] 51 (6): 603–608. 11498928

[52] Virgo BB, Bellward GD (1974) Serum progesterone levels in the pregnant and postpartum laboratory mouse. Endocrinology 95 (5): 1486–1490. 4473330 10.1210/endo-95-5-1486

[53] Schreiber JR, Hsueh AJ, Baulieu EE (1983) Binding of the anti-progestin RU-486 to rat ovary steroid receptors. Contraception 28 (1): 77–85. 6627946 10.1016/s0010-7824(83)80008-0

[54] Rohe HJ, Ahmed IS, Twist KE, Craven RJ (2009) PGRMC1 (progesterone receptor membrane component 1): a targetable protein with multiple functions in steroid signaling, P450 activation and drug binding. Pharmacology & therapeutics 121 (1): 14–19. 18992768 10.1016/j.pharmthera.2008.09.006 PMC2659782

[55] Kaluka D, Batabyal D, Chiang B, Poulos TL, Yeh S (2015) Spectroscopic and mutagenesis studies of human PGRMC1. Biochemistry 54 (8): 1638–1647. 10.1021/bi501177e 25675345 PMC4533898

[56] Meyer C, Schmid R, Schmieding K, Falkenstein E, Wehling M (1998) Characterization of high affinity progesterone-binding membrane proteins by anti-peptide antiserum. Steroids 63 (2): 111–116. 9516722 10.1016/s0039-128x(97)00143-8

[57] Cahill MA (2007) Progesterone receptor membrane component 1: an integrative review. The Journal of steroid biochemistry and molecular biology 105 (1–5): 16–36. 17583495 10.1016/j.jsbmb.2007.02.002

[58] Montano MM, Welshons WV, vom Saal F S (1995) Free estradiol in serum and brain uptake of estradiol during fetal and neonatal sexual differentiation in female rats. Biology of reproduction 53 (5): 1198–1207. 8527526 10.1095/biolreprod53.5.1198

[59] Rettew JA, McCall SH, Marriott I (2010) GPR30/GPER-1 mediates rapid decreases in TLR4 expression on murine macrophages. Molecular and cellular endocrinology 328 (1–2): 87–92. 10.1016/j.mce.2010.07.017 20654686

[60] Tran Q, Vermeer M (2014) Biosensor-based approach identifies four distinct calmodulin-binding domains in the G protein-coupled estrogen receptor 1. PloS one 9 (2): e89669 10.1371/journal.pone.0089669 24586950 PMC3931812

[61] Wesson DW, Keller M, Douhard Q, Baum MJ, Bakker J (2006) Enhanced urinary odor discrimination in female aromatase knockout (ArKO) mice. Hormones and behavior 49 (5): 580–586. 16448653 10.1016/j.yhbeh.2005.12.013 PMC2263132

[62] Cherian S, Wai Lam Y, McDaniels I, Struziak M, Delay RJ (2014) Estradiol rapidly modulates odor responses in mouse vomeronasal sensory neurons. Neuroscience 269: 43–58. 10.1016/j.neuroscience.2014.03.011 24680884 PMC4270699

[63] Moffatt CA (2003) Steroid hormone modulation of olfactory processing in the context of socio-sexual behaviors in rodents and humans. Brain research. Brain research reviews 43 (2): 192–206. 14572914 10.1016/s0165-0173(03)00208-x

[64] Caruso S, Grillo C, Agnello C, Maiolino L, Intelisano G, Serra A (2001) A prospective study evidencing rhinomanometric and olfactometric outcomes in women taking oral contraceptives. Human reproduction (Oxford, England) 16 (11): 2288–2294. 10.1093/humrep/16.11.2288 11679506

