# Supplementary material for: Modulatory Effects of Sex Steroids Progesterone and Estradiol on Odorant Evoked Responses in Olfactory Receptor Neurons
Source: PLoS One. 2016 Aug 5;11(8):e0159640. doi: 10.1371/journal.pone.0159640 (PMC4975405; doi:10.1371/journal.pone.0159640)
Supplement: S1 Fig — Gel electrophoresis of the amplified PCR products from cDNA samples of female murine OE. Used primer pairs were intron-spanning excluding gDNA amplified products. (DOCX) [file pone.0159640.s001.docx]

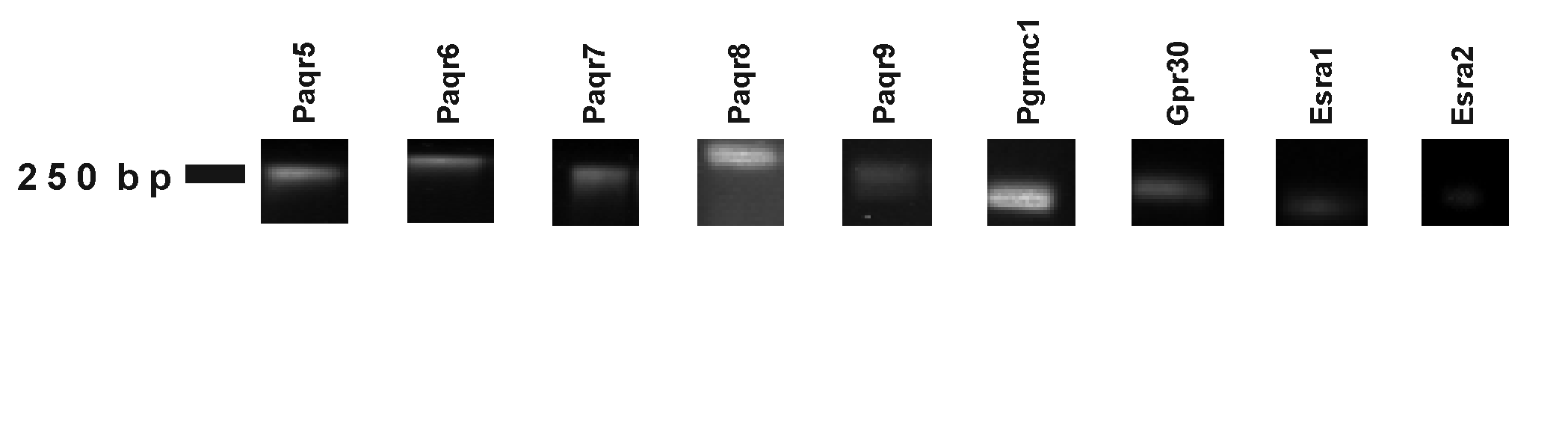


**S1 Fig:** RT-PCR analysis of GPCRs obtained from RNA-Seq. Gel electrophoresis of the amplified PCR products from cDNA samples of female murine OE. Used primer pairs were intron-spanning excluding gDNA amplified products.
