# Supplementary material for: Modulatory Effects of Sex Steroids Progesterone and Estradiol on Odorant Evoked Responses in Olfactory Receptor Neurons
Source: PLoS One. 2016 Aug 5;11(8):e0159640. doi: 10.1371/journal.pone.0159640 (PMC4975405; doi:10.1371/journal.pone.0159640)
Supplement: S2 Fig — Protein expression was assessed by staining coronal sections of OMP-GFP mice with anti-Pgrmc1. Left: GFP fluorescence (green) served to identify mature ORNs. Center: Pgrmc1 immunoreactivity (red) was observed in the cilia layer as indicated by white arrows. Right: Overlay of the GFP signal and antibody staining. Scale bar, 5 μm. (DOCX) [file pone.0159640.s002.docx]

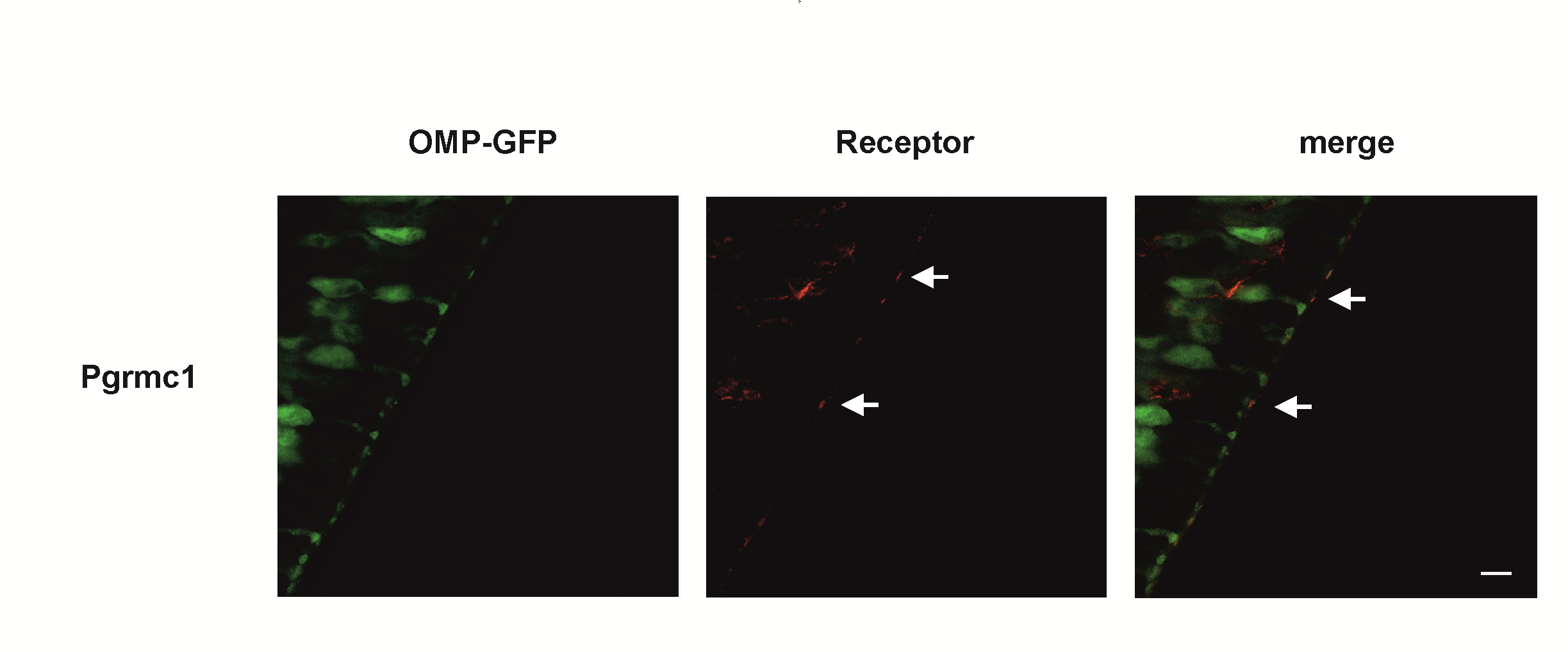


**S2 Fig:** Expression of membrane progestin receptors in the OE. Protein expression was assessed by staining coronal sections of OMP-GFP mice with anti-Pgrmc1. Left: GFP fluorescence (green) served to identify mature ORNs. Center: Pgrmc1 immunoreactivity (red) was observed in the cilia layer as indicated by white arrows. Right: Overlay of the GFP signal and antibody staining. Scale bar, 5 µm.
