# Supplementary material for: Modulatory Effects of Sex Steroids Progesterone and Estradiol on Odorant Evoked Responses in Olfactory Receptor Neurons
Source: PLoS One. 2016 Aug 5;11(8):e0159640. doi: 10.1371/journal.pone.0159640 (PMC4975405; doi:10.1371/journal.pone.0159640)
Supplement: S3 Fig — Left: GFP fluorescence (green) served to identify mature ORNs. Center: Only a low background staining is observed using the secondary antibody. Right: Overlay of the GFP signal and antibody staining. Scale bar, 5 μm. (DOCX) [file pone.0159640.s003.docx]

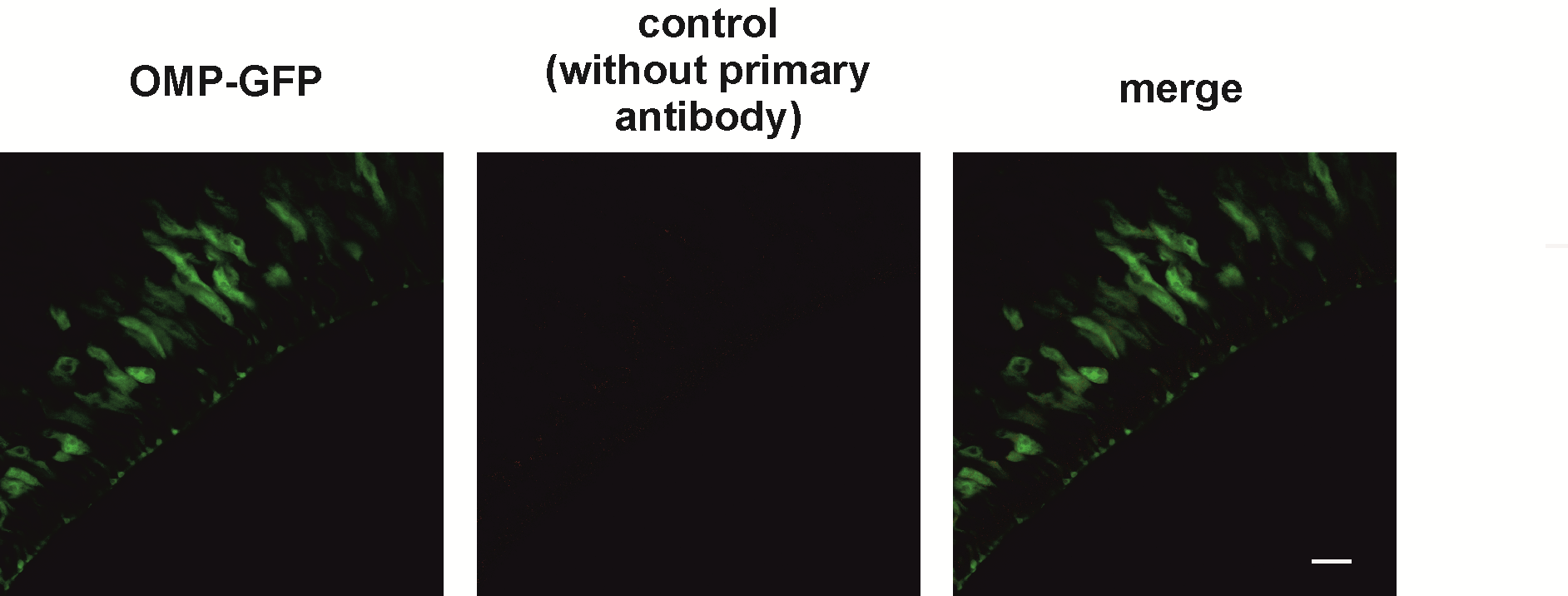


**S3 Fig:** Control staining of coronal sections of OMP-GFP mice with the secondary antibody. Left: GFP fluorescence (green) served to identify mature ORNs. Center: Only a low background staining is observed using the secondary antibody. Right: Overlay of the GFP signal and antibody staining. Scale bar, 5 µm.
