# Supplementary material for: Modulatory Effects of Sex Steroids Progesterone and Estradiol on Odorant Evoked Responses in Olfactory Receptor Neurons
Source: PLoS One. 2016 Aug 5;11(8):e0159640. doi: 10.1371/journal.pone.0159640 (PMC4975405; doi:10.1371/journal.pone.0159640)
Supplement: S1 Table — To assess sex specific differences in the expression of steroid receptors in female (fOE) and male OE (mOE), we used a cuffdiff analysis as previously described [36]; however, no sex-specific expression patterns were found. (DOCX) [file pone.0159640.s004.docx]

| **Gene** | **sample_1** | **sample_2** | **status** | **log2(fold_change)** | **p_value** | **significant** |
| --- | --- | --- | --- | --- | --- | --- |
| **Paqr5** | fOE | mOE | OK | 1.33 | 0.08 | no |
| **Paqr6** | fOE | mOE | OK | -0.14 | 0.76 | no |
| **Paqr7** | fOE | mOE | OK | 0.24 | 0.68 | no |
| **Paqr8** | fOE | mOE | OK | 0.27 | 0.54 | no |
| **Paqr9** | fOE | mOE | OK | -0.27 | 0.50 | no |
| **Pgr** | fOE | mOE | NOTEST | -0.53 | 1.00 | no |
| **Pgrmc1** | fOE | mOE | OK | 0.31 | 0.50 | no |
| **Pgrmc2** | fOE | mOE | OK | 0.30 | 0.44 | no |
| **Esr1** | fOE | mOE | NOTEST | -0.55 | 1.00 | no |
| **Esr2** | fOE | mOE | NOTEST | -0.43 | 1.00 | no |
| **Gpr30** | fOE | mOE | OK | -0.05 | 0.96 | no |

**S1 Table:** To assess sex specific differences in the expression of steroid receptors in female (fOE) and male OE (mOE), we used a cuffdiff analysis as previously described [36]; however, no sex-specific expression patterns were found.
